# SARS-CoV-2 nucleocapsid protein-specific monoclonal antibodies as tools for studying its antigenic structure and interaction with host cells

**DOI:** 10.1038/s41598-026-40984-8

**Published:** 2026-02-28

**Authors:** Agnė Rimkutė, Martynas Simanavičius, Indrė Dalgėdienė, Evaldas Čiplys, Donata Hoffmann, Kerstin Wernike, Vytautė Starkuvienė-Erfle, Aurelija Žvirblienė, Indrė Kučinskaitė-Kodzė

**Affiliations:** 1 Institute of Biotechnology, Life Sciences Center, Vilnius University, Vilnius, Lithuania; 2 Institute of Diagnostic Virology, Friedrich-Loeffler-Institut, Greifswald-Insel Riems, Germany; 3 Institute of Biosciences, Life Sciences Center, Vilnius University, Vilnius, Lithuania; 4 BioQuant, Heidelberg University, Heidelberg, Germany

**Keywords:** SARS-CoV-2, Nucleocapsid protein, Monoclonal antibodies, Immunodominant epitopes, Immunology, Nucleoproteins, Viral proteins, Antibody generation

## Abstract

Diagnostics and prevention of COVID-19 are essential for controlling the spread of the virus and reducing mortality rates. As SARS-CoV-2 surface proteins are susceptible to mutations, the nucleocapsid protein (NP) with its highly conserved gene sequence is an attractive target for studying virus-host interactions. NP plays a key role in the coronavirus life cycle, modulating viral RNA packaging, transcription, and assembly. In addition, its abundant expression during infection makes it a valuable diagnostic marker. NP is involved in modulating the host’s innate immunity; however, the cellular mechanisms of its pathogenicity are not yet fully understood. This study developed and characterized murine monoclonal antibodies (MAbs) specific to the SARS-CoV-2 NP to investigate its antigenic regions and utilize the MAbs in virus-detecting systems or cellular NP blocking assays. The MAbs showed cross-reactivity with Omicron NP, recognizing epitopes within functionally active domains. They also identified NP in SARS-CoV-2-infected cells, supporting their feasibility in future immunoassays. Additionally, the ability to inhibit NP-cell interaction was assessed, with MAbs 4B3, 7F10, 16D9, and 18A8 found to reduce NP internalization. Overall, this study provides well-characterized tools for investigating SARS-CoV-2 antigenicity and pathogenicity and demonstrates the functional potential of the generated MAbs in studying NP-mediated host cell interactions.

**Supplementary Information:**

The online version contains supplementary material available at 10.1038/s41598-026-40984-8.

## Introduction

In the last two decades, coronaviruses have caused three major outbreaks of severe respiratory diseases^1–3^. The emergence of novel SARS-CoV-2 in late 2019 has affected nearly 800 million people worldwide and imposed a huge socio-economic burden around the globe^4^. Although vaccines were introduced^5^ and helped to control severe disease and mortality rates, SARS-CoV-2 continues to spread^6^. Genetic mutations in SARS-CoV-2 variants significantly affect the immunogenicity of viral proteins, facilitating immune escape. Despite the efficacy of approved vaccines and other therapeutics, immune-evasive variants have reduced protection against the virus^7^. The emergence of novel virus variants can lead to breakthrough infections^8^ highlighting the need for continued research in COVID-19 diagnostics, prevention, and treatment, as well as fundamental studies investigating the molecular mechanisms of the virus.

The extensively studied SARS-CoV-2 spike protein, which mediates viral entry^9^, is the primary target for vaccines and the development of neutralizing monoclonal antibodies (MAbs)^10^. However, other major structural coronavirus proteins also play a significant role in the virus life cycle and the progression of infection. Among them is the SARS-CoV-2 nucleocapsid protein (NP), which is an important determinant of virulence and pathogenicity, responsible for binding and packaging viral RNA^11^. Structurally, NP contains an N-terminal domain (NTD) and a C-terminal domain (CTD), flanked by the N-arm and C-tail, respectively. NTD facilitates RNA binding, while CTD is essential for RNA binding and dimerization—a critical step in RNA packaging and viral assembly. These domains are connected by a central linker region (LKR), which includes a serine/arginine (SR)-rich motif^12^. Cytoplasmic kinases rapidly phosphorylate the SR-rich region at the beginning of infection, enhancing NP’s affinity for viral RNA and promoting efficient genome packaging^13^. Overall, NP is essential for binding RNA and organizing it into a helical ribonucleoprotein complex, regulating RNA synthesis during replication and transcription, and modulating virus particle assembly^14^. Importantly, NP evolves at a significantly slower rate than the spike protein, with low mutation frequencies observed across variants of concern. Numerous SARS-CoV-2 variants, including B.1.1.7, B.1.351, P.1, B.1.617.2, and BA.1, emerged during 2020–2021, driven largely by mutations in the spike protein. Although several NP mutations, such as P13L, Δ31-33, R203K, G204R, and S413R, have been consistently detected in Omicron sublineages BA.4, BA.5, XBB.1.5, JN.1, KP.2, and LP.8.1 (outbreak.info^15^, last updated August 10, 2025). Of note, the G204R and G204K substitutions, found in B.1.1.7, P.1, and B.1.1.529 lineages, have been reported to enhance viral replication and transmission efficiency^16^. Nevertheless, the overall sequence of NP has remained highly conserved, showing > 98% sequence identity across these variants. Moreover, NP shares ~ 90% sequence similarity with the NP of SARS-CoV, the virus responsible for the 2002–2004 SARS outbreak^17,18^, further underscoring its evolutionary conservation.

Apart from its pathogenic effects, SARS-CoV-2 NP serves as an excellent serodiagnostic marker for COVID-19^19^. Due to its high immunogenicity, NP elicits a robust humoral response even in mild or asymptomatic cases. Moreover, it is one of the most abundantly expressed proteins during coronavirus infection^20^. It is estimated that a single virion contains 10 times more copies of NP than copies of spike protein, making NP an ideal target for the development of antigen-detecting rapid SARS-CoV-2 tests using specific MAbs^21,22^. Although NP-based diagnostic assays show considerable variability in sensitivity depending on the timing of sample collection; however, studies demonstrate that the CTD of NP is the most immunogenic, suggesting that focusing on this domain could enhance the performance of serological tests^23^. Nevertheless, other multi-systemic analyses indicate that B-cell immunodominant epitopes of NP are likely distributed throughout the entire protein^17,24,25^.

Besides its role in the viral life cycle, NP is also involved in host cellular processes. Early in the infection, it suppresses innate immunity to support viral replication^26^. Compared to other common pathogenic respiratory viruses such as influenza A virus, human parainfluenza virus type 3, or respiratory syncytial virus, SARS-CoV-2 induces lower levels of interferon (IFN) response, which contributes to its increased immunosuppressive ability^27^. Specifically, SARS-CoV-2 NP is associated with inhibiting IFN-α and IFN-β by targeting the RIG-I signaling pathway. This impaired IFN response is thought to contribute to the severity of COVID-19^28^. Additionally, NP inhibits RNA interference, disrupting the host cell’s ability to degrade viral RNA. Therefore, the suppression of RNA interference not only impairs the host’s antiviral defense but also promotes viral replication^29^. This phenomenon is similarly observed with NPs of other coronaviruses^30^. However, NP can also trigger inflammatory pathways, leading to pathological inflammation^27^. Upon the association with viral RNA, signaling kinases are recruited, thus stimulating the activation of the NF-κB signaling, releasing multiple inflammatory cytokines, such as IL-6, IL-1β, and TNF-α^31^. Studies have also proven that NP can directly interact with the NLRP3 protein, promoting the assembly and activation of the inflammasome complex^32^. Furthermore, after the NP enters the cytosol, it specifically binds to Gasdermin D, a key effector of pyroptosis responsible for forming membrane pores upon cleavage by inflammatory caspases. By blocking Gasdermin D activation, NP prevents pyroptotic cell death, allowing the virus to evade immune detection^33^.

While NP is primarily known for its role inside the host cell, studies have shown that significant amounts of NP are also found in the extracellular space^34^. Interestingly, NP of SARS-CoV-2 demonstrated the highest cell-type independent surface binding affinity among other human coronaviruses (hCoVs), suggesting significant pathogenic implications^35^. Investigations have shown that extracellular NP also contributes to inflammation by complement hyperactivation through its direct interaction and activation of the complement system protease MASP-2. The hyperactivation of the complement can significantly contribute to immune dysregulation, tissue damage, or organ dysfunction^36^. The ability of both intracellular and extracellular SARS-CoV-2 NP to suppress the host’s innate antiviral immunity while promoting inflammatory responses may explain the sudden virus spread worldwide. However, more insights regarding the molecular function of SARS-CoV-2 NP are needed.

Here, we report the development of a novel collection of murine MAbs against recombinant SARS-CoV-2 NP and their characterization by various immunoassays. This study provides new data on the antigenic structure of the SARS-CoV-2 NP and demonstrates the potential of the newly generated MAbs as valuable tools for developing COVID-19 antigen-detecting immunoassays and investigation of host–virus interactions needed for advanced SARS-CoV-2 pathogenicity research.

## Results

### Development and characterization of MAbs against SARS-CoV-2 NP

SARS-CoV-2 recombinant full-length NP and a variant with a deletion of aa 1–120 in the N-terminus (NP-ΔN) produced in *S. cerevisiae* were used to generate MAbs. A truncated NP-ΔN protein variant was chosen to increase specificity due to the high sequence homology of the N-terminal region among NPs from different coronaviruses^37^. The immunogenicity of the antigens was evaluated by indirect ELISA. After the third immunization, both NP variants were proven to be immunogenic, since they induced NP-specific antibody titer in mice (> 1:600). Hybridoma production yielded a total of nine stable cell lines, comprising two secreting antibodies against the full-length NP and seven specific for NP-ΔN. All hybridomas secreted antibodies of IgG1 subtype (κ light chain). The MAbs purified by affinity chromatography were characterized for their binding affinities using ELISA and BLI, as summarized in Table 1. All generated MAbs were found to be specific to recombinant NPs, as they did not react with irrelevant proteins in the lysate of *S. cerevisiae* (data not shown). To determine the affinity of the MAbs, the K_d_ was measured by an indirect ELISA. The Kd values with NP-ΔN ranged from 36.6 to 235 pM, with the exception of MAb 7F10, for which no binding affinity was determined due to the absence of its epitope in this truncated NP variant (further details on epitope mapping are provided below). The binding affinities of the MAbs to full-length NP ranged from 33 to 1060 pM, and from 44.1 to 4760 pM with the recombinant NP of the SARS-CoV-2 Omicron variant (BA.5) (NP_Omicron_). MAb binding kinetics to the NP_Omicron_ were further analyzed by bio-layer interferometry (BLI) using the Octet K2 system (Table 1, Fig. S1a), which directly monitors binding between immobilized antibody and soluble antigen in real time, revealing kinetic differences, unlike ELISA (sensorgrams presented in Fig. S1b). The K_d_ values obtained by BLI were higher than those measured by ELISA, ranging from 704.4 to 7273 pM. Comparison of the antibody binding characteristics to the NP_Omicron_ measured by ELISA and BLI showed that MAbs 4G6, 7F10, 1A6, 4B3, 6G11, and 12B2 displayed high to moderate affinity in both assays, whereas lower affinity was observed for MAbs 16D9 and 18A8 in ELISA and for MAb 13C10 in BLI. Notably, for MAbs 16D9 and 18A8, antigen binding was undetectable in BLI, and K_d_ values were therefore not determined (Fig. S1a).

**Table 1:**
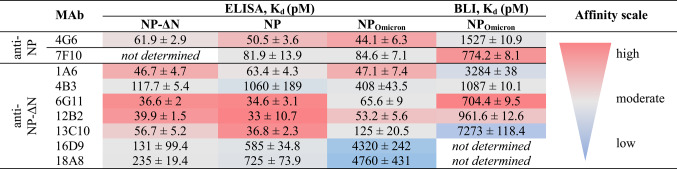
Evaluation of binding affinity of SARS-CoV-2 anti-NP MAbs by ELISA and BLI.

NP-ΔN – SARS-CoV-2 recombinant nucleocapsid protein with an N-terminal deletion.

NP – SARS-CoV-2 recombinant nucleocapsid protein.

NP_Omicron_ – SARS-CoV-2 recombinant nucleocapsid Omicron (BA.5) protein.

The results are reported as value (pM) ± SEM.

For additional characterization, the MAbs were evaluated by WB using NP, NP_Omicron_, and recombinant NPs from other hCoVs, including SARS-CoV, NL63, and OC43. All MAbs recognized the NP and NP_Omicron_, consistent with previous assays. Notably, MAbs 16D9 and 18A8 reacted weakly with the full-length NP and NP_Omicron_ but showed strong binding to a truncated version of the protein. No reactivity was observed with NL63-NP-ΔN and OC43-NP-ΔN, whereas all MAbs bound to SARS-CoV NP as detected by WB (Table 2, Fig. S2).

**Table 2:** WB analysis of SARS-CoV-2 anti-NP MAbs.

	MAb		WB with recombinant NPs					WB with viral NPs	
		NP-ΔN	NP	NP_**Omicron**_	SARS-CoV-NP	NL63-NP-ΔN	OC43-NP-ΔN	WT	XBB.1.5
anti- NP	4G6	** + **	** + **	** + **	** + **	**−**	**−**	** + **	** + **
	7F10	**–**	** + **	** + **	** + **	**−**	**−**	** + **	** + **
anti- NP-ΔN	1A6	** + **	** + **	** + **	** + **	**−**	**−**	** + **	** + **
	4B3	** + **	** + **	** + **	** + **	**−**	**−**	** + **	** + **
	6G11	** + **	** + **	** + **	** + **	**−**	**−**	** + **	** + **
	12B2	** + **	** + **	** + **	** + **	**−**	**−**	** + **	** + **
	13C10	** + **	** + **	** + **	** + **	**−**	**−**	** + /−**	** + /−**
	16D9	** + **	** + /−**	** + /−**	** + **	**−**	**−**	** + **	** + **
	18A8	** + **	** + /−**	** + −**	** + **	**−**	**−**	** + **	** + **

“ + ” – a visible band defined as a positive reaction (intensity > 2000), “ + /**−**” – weak visible band (1000 ≤ intensity < 2000), “**−**” – no band defined as a negative reaction (intensity < 1000).

**Fig. 1:**
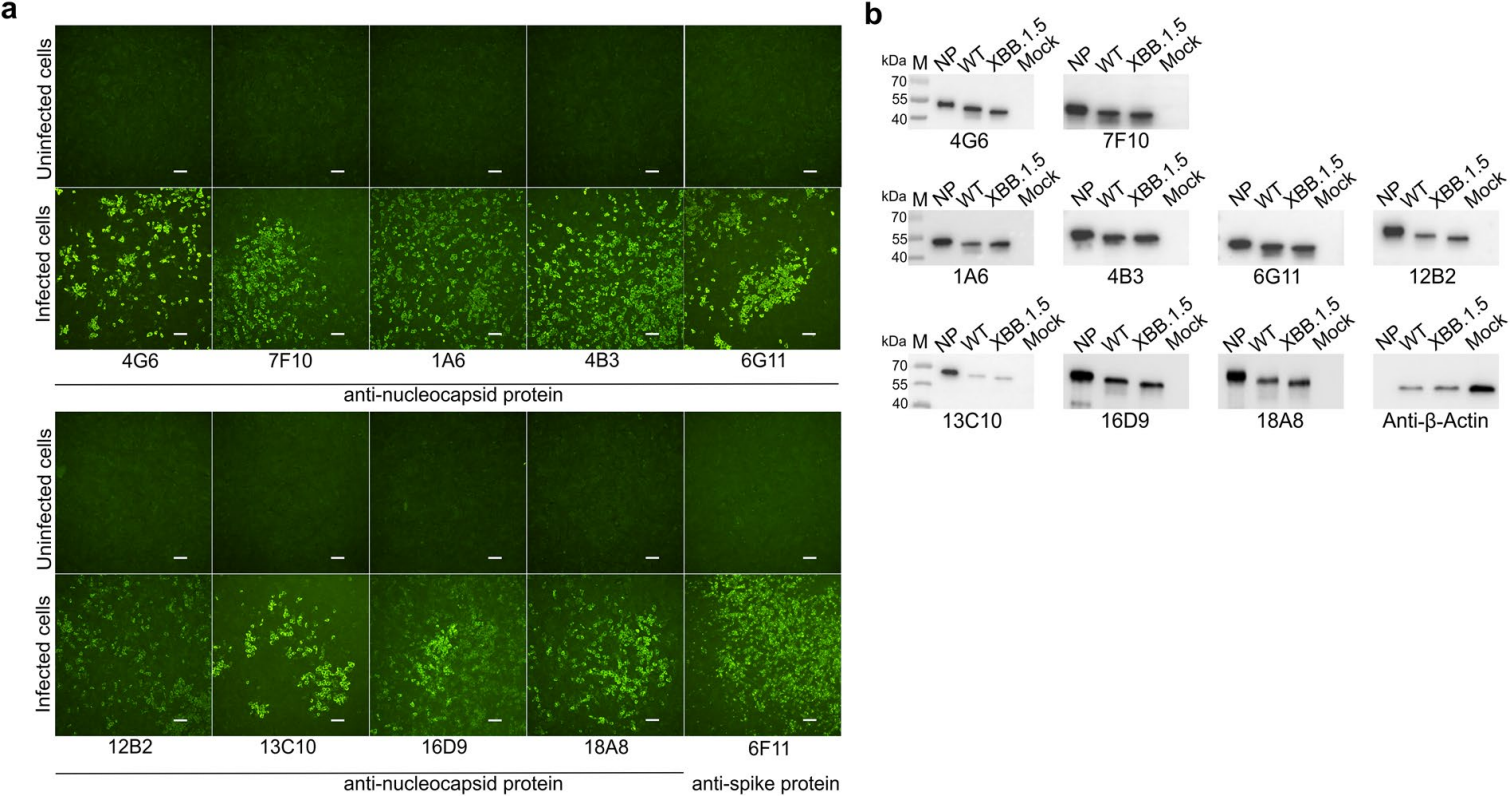
The reactivity of the MAbs raised against SARS-CoV-2 NP with natural viral nucleoprotein. (**a**) Microscopy images demonstrating the results of indirect immunofluorescence analysis of MAbs reactivity with SARS-CoV-2 wild-type virus (strain 2019_nCoV BavPat1) infected Vero E6 cells. As a control, in-house produced anti-spike MAb 6F11 (IgG1) was used. The uninfected control refers to non-infected cells stained with MAbs. The scale bar indicates 100 µm; images were taken using 4 × objective; (**b**) Western Blot analysis of anti-NP MAbs reactivity with SARS-CoV-2 wild-type virus (SARS-CoV-2 Germany/BavPat1/2020) and Omicron XBB.1.5 variant (hCoV-19/Netherlands/NH-EMC-5667/2023) infected Vero E6 cell lysates. Mock indicates lysate from non-infected cells. Original blots are presented in Supplementary Fig. S3.

MAbs reactivity was also tested with viral NP by iIFA and WB. For the iIFA, SARS-CoV-2 WT virus-infected Vero E6 cells were stained with each MAb. The results indicated that all MAbs recognize viral NP in SARS-CoV-2 infected cells (Fig. 1a). For WB analysis, lysates from Vero E6 cells infected with SARS-CoV-2 WT and Omicron XBB.1.5 variants were used. The WB results showed that all MAbs recognized the viral NP of both tested SARS-CoV-2 variants (Table 2, Fig. 1b), despite MAbs 16D9 and 18A8 showing only weak reactivity with the recombinant full-length SARS-CoV-2 NP.

The results indicate that all nine generated hybridoma cell lines secrete MAbs, displaying high affinity and specificity against SARS-CoV-2 NP. These MAbs recognize natural SARS-CoV-2 NP and cross-react with SARS-CoV-2 Omicron variant, including XBB.1.5, which differs from WT by five NP mutations^38^.

To extend the characterization of the generated MAbs, their performance was evaluated in a sandwich ELISA format. All antibodies were conjugated with HRP and tested in a competitive ELISA to identify non-competing pairs recognizing distinct epitopes. The analysis revealed overlapping binding sites for MAbs 4G6 and 6G11, as well as for 16D9 and 18A8. Various MAb combinations were then examined in a sandwich ELISA format, and the highest sensitivity was achieved with MAb 6G11 as a capture antibody and MAb 12B2-HRP as a detection antibody. The optimized assay showed a strong correlation between NP or NP_Omicron_ concentrations and OD_450_ values (R^2^ = 0.9986 and 0.9959, respectively) (Fig. S4). These results demonstrate the diagnostic potential of the generated MAbs and provide a basis for further assay development.

### Mapping of epitopes recognized by the MAbs

**Fig. 2:**
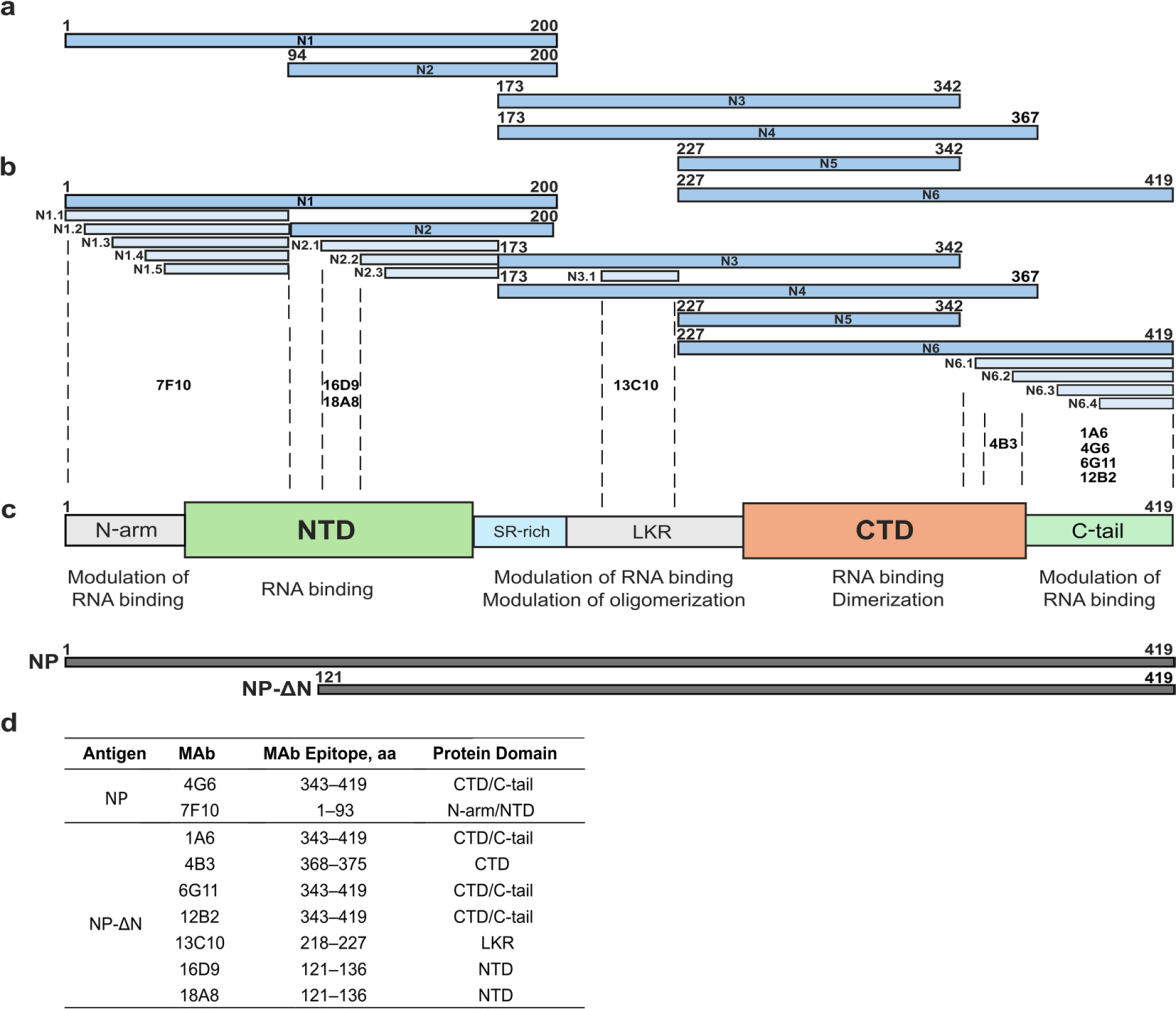
The schematic overview of the generated overlapping SARS-CoV-2 NP fragments and MAb recognition sites according to functional protein domains. (**a**) Fragments N1-N6, colored in blue; (**b**) truncated N1-N6 protein fragments, colored in light blue; (**c**) SARS-CoV-2 NP domain organization. The full-length NP and truncated NP protein variants used in this study are colored in grey; (**d**) the predicted epitopes of the developed MAbs using overlapping NP fragments.

To localize the binding sites of the MAbs within the WT SARS-CoV-2 NP, six overlapping recombinant protein fragments were produced in the *E. coli* Rosetta-gami 2(DE3) strain. The reactivity of MAbs with each NP fragment was assessed by WB. A schematic representation of the overlapping NP fragments used for epitope mapping is shown in Fig. 2a. WB analysis revealed that MAb 7F10 recognizes only the N1 fragment, suggesting its epitope is within aa 1–93 of NP. MAbs 16D9 and 18A8 reacted with both N1 and N2 fragments. Since these MAbs were raised against a truncated NP variant (NP-ΔN), their epitopes were mapped within the aa 121–172 region. MAb 13C10 recognized only N3 and N4 fragments, indicating that its epitope is between aa 201–227. MAbs 1A6, 4G6, 6G11, and 12B2 reacted with N4 and N6 fragments, suggesting their binding site is in the region between aa 343–419, while MAb 4B3 recognized only N6, indicating that its binding site is between aa 368–419.

For fine epitope mapping, the previously described overlapping fragments were truncated into 13 smaller segments according to the determined MAb binding sites (Fig. 2b). Truncated NP fragments were expressed in the *E. coli* Tuner(DE3) strain. The results of WB analysis narrowed down the epitope of MAbs 16D9 and 18A8 to the NP sequence spanning aa 121–136, as they reacted exclusively with the N2.1 fragment. Similarly, the epitope recognized by MAb 13C10 was localized to aa 218–227, since it reacted with the fragment N3.1. The binding site of MAb 4B3 was mapped to the aa 368–375 region, as it reacted with fragments N6.1 and N6.2. Other MAb epitopes were not identified more precisely. The summary of the epitope mapping results is provided in Fig. 2d (The original blots are provided in Supplementary Fig. S5, and their summary is included in Supplementary Table S3). The analysis indicated that MAbs 16D9, 18A8, and partially 7F10 bind within the RNA binding domain of NP. Likewise, the binding sites of MAbs 1A6, 4G6, 6G11, and 12B2 are located within the C-terminal domain of NP, which is involved in both RNA binding and protein dimerization. These findings suggest that eight out of nine developed MAbs recognize the epitopes located in functionally active regions of NP molecule (Fig. 2c).

**Fig. 3:**
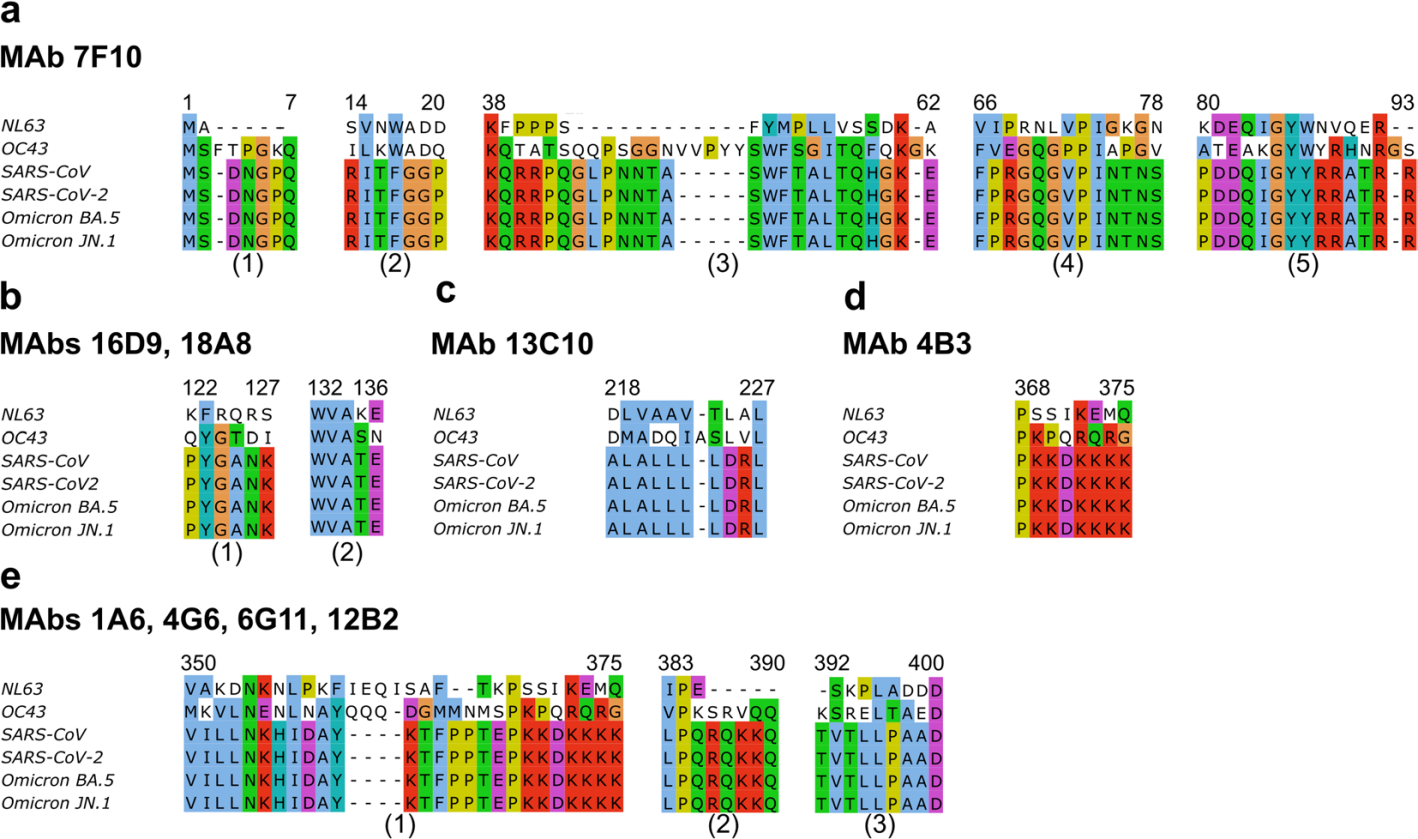
The sequence alignments of NP of hCoV NL63 (Q6Q1R8), hCoV OC43 (P33469), SARS-CoV (P59595), SARS-CoV-2 WT (P0DTC9), SARS-CoV-2 Omicron BA.5 (UOZ45812.1) and SARS-CoV-2 Omicron JN.1 (XLG88095.1), indicating potential binding sites of the MAbs. (**a**) MAb 7F10, (**b**) MAbs 16D9, 18A8, (**c**) MAb 13C10, (**d**) MAb 4B3, (**e**) MAbs 1A6, 4G6, 6G11, 12B2. The sequences were aligned using the multiple sequence alignment program MAFFT and visualized with Jalview.

The results of both epitope mapping and cross-reactivity assays were used for the following predictive analysis of potential MAb epitopes. Multiple sequence alignments of NPs of different hCoVs, including the recently emerged SARS-CoV-2 Omicron JN.1 variant, were performed using MAFFT program. Since it was determined that none of the MAbs reacted with NPs of NL63 or OC43, and their binding sites are in regions that remain unchanged during virus evolution (identified mutations listed in Table S4), the predictive analysis identified only a few potential binding sites for MAbs. The epitope of MAb 7F10 consists of five distinct sequence regions between aa 1–7, aa 14–20, aa 38–32, aa 66–78, and aa 80–94 (Fig. 3a). The sequence comprised of aa 122–127 and aa 132–136 represents a potential epitope for MAbs 16D9 and 18A8 (Fig. 3b). The binding site of MAb 13C10 is between aa 218–227, while 4B3 epitope is predicted to be between aa 368–375 (Fig. 3c, d, respectively). Additionally, MAbs 1A6, 4G6, 6G11, and 12B2 recognize an epitope within the regions spanning aa 350–376, aa 383–390, or aa 392–400 (Fig. 3e).

### Investigation of MAb-recognized and naturally occurring epitopes of SARS-CoV-2 NP

To investigate whether the naturally occurring epitopes recognized by human antibodies specific to SARS-CoV-2 NP align with those targeted by generated MAbs, a MaE was performed. NP and NP_Omicron_ were printed in the microarray at the bottom of the 96-well plate. Each anti-NP MAb was applied to the wells. To assess competition for epitope binding, fifteen serum specimens confirmed as SARS-CoV-2 anti-NP IgG positive by the Coronavirus IgG MaE Test (UAB Imunodiagnostika, Lithuania) were added. Additionally, three serum specimens, confirmed as negative using this test, were used as negative controls. The weak reactivity of MAbs 16D9 and 18A8 with recombinant full-length SARS-CoV-2 NP in WB (Fig. S2) was also consistently observed in the MaE assay. Consequently, MAbs 16D9 and 18A8 were excluded from further MaE analysis. The results of MaE demonstrated that the generated NP-specific MAbs do not interfere with the binding of antibodies present in convalescent sera (Table S5, Fig. S6). This suggests that the antigenic epitopes targeted by the murine-derived anti-NP MAbs may be either distinct from or not fully overlapping with those recognized by naturally occurring human antibodies, and that a single MAb-epitope interaction might not be sufficient to inhibit binding of polyclonal serum antibodies to the multiple epitopes.

### MAb capacity to inhibit the cellular entry of SARS-CoV-2 NP

**Fig. 4:**
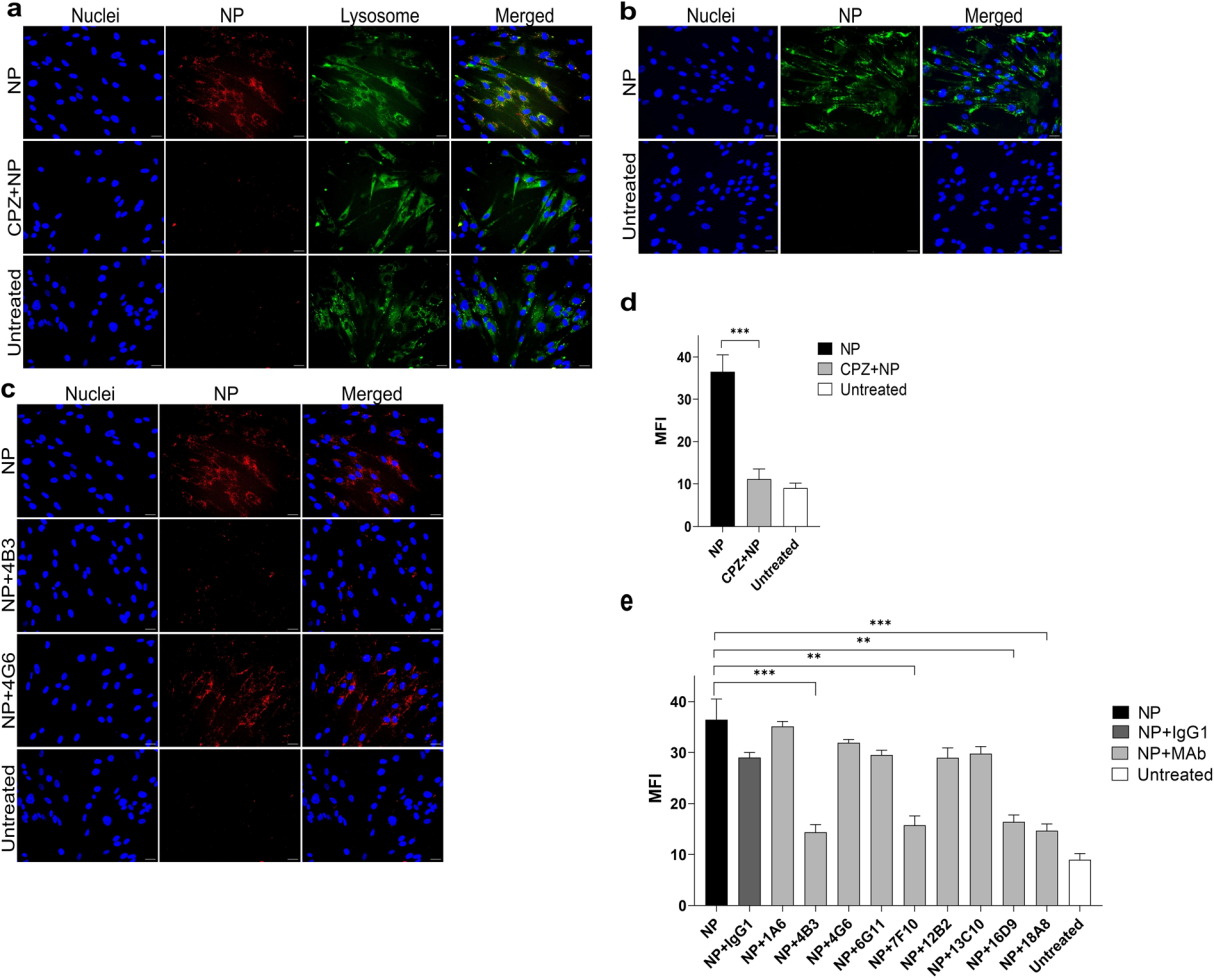
The analysis of SARS-CoV-2 NP entry to the intracellular space of IMR-90 cells. (**a**) Fluorescence microscopy analysis of endocytosis of NP_Omicron_-Deep Red (red): alone, pretreated with CPZ, and negative control without the protein. The cells were also stained with nuclear stain Hoechst 33342 (blue) and lysosomal marker (green); (**b**) – iIFA of NP_Omicron_-Deep Red treated cells stained with SARS-CoV-2 NP-specific 6G11 MAb (green) compared to negative control with only secondary antibody. The cells were also stained with Hoechst 33342 (blue); (**c**) – fluorescence microscopy analysis of cell entry of NP_Omicron_-Deep Red (red): alone and in combination with anti-SARS-CoV-2 NP MAbs 4B3 and 4G6, compared to the negative control without the protein. The cells were also stained with nuclear stain Hoechst 33342 (blue); (**d**) MFI of NP_Omicron_-Deep Red alone and CPZ + NP_Omicron_-Deep Red (n = 3). The bar graph represents fluorescence of NP from three independent experiments, mean ± SEM; Student’s t-test, ***p < 0.001; e – MFI calculated of each NP_Omicron_-Deep Red + MAb combination (n = 3). As an isotype control, SARS-CoV-2 NP nonreactive IgG1 MAb was used. MFI calculated using ImageJ. The scale bar indicates 100 µm, images were taken using 20 × objective. The bar graph represents the fluorescence of NP from three independent experiments, mean ± SEM; Student’s t-test, **p < 0.01, ***p < 0.001. NP_Omicron_-Deep Red is referred to as NP in Fig. 4.

It is known that recombinant SARS-CoV-2 NP can bind directly to the cell surface and enter the cell by receptor-mediated endocytosis^39^ playing an additional role in SARS-CoV-2 pathogenesis. Given that some of the generated MAbs recognize epitopes within functionally active regions of the NP, a blocking assay was performed to determine whether these MAbs could prevent the NP from entering the lung cells in vitro. To investigate NP internalization, NP_Omicron_ was chosen because WT strain is no longer prevalent, and more recent variants like Omicron BA.5 have become more dominant. For this, pHrodo™ Deep Red TFP Ester-labeled NP_Omicron_ (NP_Omicron_-Deep Red) was incubated with IMR-90 cells. As a control, the endocytosis inhibitor CPZ was used. Lysosomal localization of NP_Omicron_-Deep Red was assessed using the lysosome marker LysoBrite™ Green. The results showed that after 4 h, NP_Omicron_-Deep Red is detected intracellularly, with the localization patterns strongly resembling with lysosomes (Fig. 4a). Furthermore, CPZ treatment significantly reduced NP_Omicron_-Deep Red intracellular accumulation (Fig. 4a, d). To further confirm the localization of NP, iIFA was performed. NP_Omicron_-Deep Red-treated IMR-90 cells were fixed and stained with SARS-CoV-2 NP-specific MAb 6G11. Fluorescence microscopy analysis confirmed NP intracellular entry (Fig. 4b), compared to the negative control without the NP.

For the MAb blocking assay, NP_Omicron_-Deep Red was preincubated with each MAb (molar ratio 1:2) before being added to IMR-90 cells for 4 h. As an isotype control, an irrelevant IgG1 subtype MAb specific to β-lactamase was used. Compared to NP_Omicron_-Deep Red alone, fluorescence intensity analysis revealed differences when the fluorescent NP was preincubated with MAbs 4B3 and 4G6 (Fig. 4c). Further analysis demonstrated that MAbs 4B3, 7F10, 16D9, and 18A8 significantly reduce NP_Omicron_-Deep Red cell entry (Fig. 4e). In contrast, MAbs 1A6, 4G6, 6G11, 12B2, and 13C10 did not show the same effect. These results align with the analysis of the predictive MAb binding sites, as MAbs 1A6, 4G6, 6G11, and 12B2 likely recognize an adjacent epitope, while MAb 13C10 recognizes a distinct epitope.

## Discussion

Despite the vaccines have significantly reduced the impact of the COVID-19 pandemic, SARS-CoV-2 remains in circulation due to its rapid transmission route and the continuous emergence of new variants. Therefore, there is a great demand for research on the antigenic and structural characteristics of SARS-CoV-2, as well as its interaction with the host immune system^40,41^. SARS-CoV-2 NP is a multifunctional RNA-binding viral antigen, responsible for packing virus RNA into a helical capsid structure. As the most abundantly expressed protein during the coronavirus infection and one that is less prone to genetic mutations, NP serves as a valuable component for SARS-CoV-2 molecular research^42^.

COVID-19 patients show stronger NP-specific immune responses compared to other SARS-CoV-2 structural antigens^43^. However, it remains unclear whether antibodies against NP play a direct antiviral role during infection, as NP is not involved in virus entry. Recent studies have shown that, although NP-specific antibodies cannot block the virus from entering host cells, they may still contribute to controlling SARS-CoV-2 infection and disease progression^44^. In this study, novel murine MAbs directed against SARS-CoV-2 NP were generated and characterized for their antigen recognition, epitope specificity, and functional properties, with the aim of providing tools for studies of NP pathogenesis. As a result, nine hybridomas secreting IgG1 subtype (κ light chain) anti-SARS-CoV-2 NP MAbs were developed, demonstrating high affinity and specificity to SARS-CoV-2 NP. Affinity was estimated by measuring apparent K_d_ by ELISA and exact K_d_ by BLI kinetic assay. The values obtained by these assays while testing the same antibody differ. This difference is due to experimental procedures. In the BLI assay, the MAb is captured on the sensor as the ligand, and the antigen, as the analyte in solution, binds to the immobilized antibody. In the indirect ELISA, the antigen is immobilized on the surface, and the antibody binds the antigen. Furthermore, the antibody-antigen complex in ELISA is detected by the secondary antibody. In BLI, the real-time measurements are performed, while ELISA is an endpoint assay. These experimental set-up differences should have influenced the affinity evaluation results. BLI results indicate that seven out of nine MAbs are high-affinity binders as their K_d_s are in the nanomolar range. For the remaining two MAbs (16D9 and 18A8), no measurable binding was observed by BLI. Notably, both MAbs efficiently recognized viral NP in SARS-CoV-2-infected cells by iIFA and WB, indicating that they can detect native NP. Epitope mapping showed that 16D9 and 18A8 target a region between aa 121–136, which is present in the NP_Omicron_ used in BLI. However, both MAbs reacted strongly with a truncated NP (aa 121–419) in WB, while showing only weak binding to the full-length NP and NP_Omicron_ in the same assay, suggesting the absence of detectable binding in BLI likely reflects conformational masking of this region in the full-length NPs. Despite this, all nine MAbs, including 16D9 and 18A8, which exhibited reduced signal intensity, were able to detect NP_Omicron_ by ELISA and WB, supporting their broad utility. The mapped epitopes do not overlap with the five Omicron BA.5 NP mutations (P13L, ERS31-33del, R203K, G204R, S413R)^45^**,** nor with the Q229K^46^ mutation present in more recent variants. This implies that the binding sites remain conserved, highlighting the potential of these MAbs for reliable immunodetection across circulating SARS-CoV-2 strains.

To further define the antigenic landscape of NP, a detailed analysis of MAb epitopes was carried out. Identifying epitope localization is essential for the accuracy of antibody-based diagnostics, as well as for molecular research and investigations to better understand host–pathogen interactions. The analysis proved that MAb recognition sites are distributed across the entire length of NP. Two MAbs, 16D9 and 18A8, targeted to the RNA-binding domain, three MAbs, 7F10, 13C10, and 4B3, recognized epitopes within an intrinsically disordered regions, and four other MAbs, 1A6, 4G6, 6G11, and 12B2, recognized epitopes overlapping with NP dimerization domain. These characteristics make the NP-specific MAbs valuable molecular tools for SARS-CoV-2 research, as binding sites of the MAbs are located within the functional domains of the NP. The RNA-binding domain is crucial for the formation of the viral ribonucleoprotein complex and genome replication, and the molecules that block this domain have been confirmed as potentially effective antiviral tools^46^. Since the NP lacks RNA sequence binding specificity, RNA sequences not present in the SARS-CoV-2 genome could potentially inhibit NP-viral RNA binding. A recent study demonstrated that guanosine 12-mer effectively inhibited RNA-NP interactions^47^. Small molecules, such as the antibiotic ceftriaxone, have also been shown to inhibit ribonucleoprotein complex formation by competing with viral RNA for binding to the N-NTD^48^. Peng and colleagues identified that S51, F53, Y109, Y111 and R149 are key residues of SARS-CoV-2 NTD involved in protein-RNA interactions^46^. Given these findings, constructs based on NP-specific MAbs which recognize epitopes targeting this region may also inhibit NP-RNA interactions, further supporting the potential of this domain for intervention strategies at the molecular level. As mentioned, SARS-CoV-2 NP CTD is a critical domain for both viral RNA binding and oligomerization. Wu and colleagues also demonstrated that the CTD region of SARS-CoV-2 NP is essential for both the liquid–liquid phase separation of the NP and its ability to regulate NF-κB, since the mutations in this region can impair these phase separation processes and affect the NP’s ability to regulate NF-κB signaling, influencing host cell responses, including immune system modulation and inflammation^49^. The NTD and CTD are connected and flanked by highly disordered linker regions, whose dysregulation can impair NP function and viral assembly. The central intrinsically disordered region, particularly the leucine/glutamine-rich subdomain (aa 210–246), is critical for NP-mediated phase separation and the formation of RNA–protein condensates^50^. Additionally, the carboxy terminus in the C-tail interacts with the ectodomain of the membrane protein, and together, they contribute to virion assembly, highlighting its important role in virion packaging^51^. These findings provide insight that MAbs targeting specific NP epitopes could be valuable for investigating the functional regions of SARS-CoV-2 NP and may potentially aid in the development of novel antiviral inhibitors or vaccine strategies.

Although the anti-NP MAbs described here recognize functional domains of the NP, these epitopes may differ from those most frequently targeted by naturally occurring human antibodies. The MaE results showed that none of the MAbs were able to inhibit the binding of natural SARS-CoV-2 antibodies from convalescent sera positive for anti-NP IgG to either recombinant NP or NP_Omicron_. These differences may arise from fundamental variations in the immune systems of different species. For instance, the B cell repertoire in mice differs from that in humans, which may lead to species-specific preferences in epitope targeting^52^. This is supported by proteome peptide microarray screening, which identified several immunoreactive regions on NP. Musico and colleagues, for example, screened 50 serum samples from COVID-19 patients and highlighted an IgG epitope within aa 155–171 of NP that showed good diagnostic performance^53^. Similarly, another study analyzing over 80 human blood specimens from active and post-COVID-19 infections detected an NP epitope spanning aa 156–176^54^. None of the MAbs described in this study recognize the mentioned region of NP. In contrast, these anti-NP MAbs appear to target epitopes similar to those recognized by other murine-derived MAbs reported in the literature^55^. However, several studies have mapped immunodominant regions of NP that are located within or near the epitopes recognized by the MAbs described here. The study done by Zamecnik et al. (2020) identified four potential NP epitopes using a programmable phage display library combined with microarray screening of COVID-19 patient sera. Three aa regions, aa 134–171, aa 210–247, aa 362–399, fully or partially include epitopes recognized by MAbs 16D9 and 18A8, 13C10, and 1A6, 4G6, 4B3, 6G11, 12B2, respectively^56^. These findings are complemented by a study by Wen and colleagues, in which the mapping of epitopes of COVID-19 patients derived MAbs revealed potential binding sites aa 46–174, and aa 245–364. These aa regions also include most of the epitopes recognized by the generated MAbs^57^ . While these studies provide valuable insight into the antigenic landscape of NP, many mapped regions rely heavily on bioinformatic predictions, which, though informative, are not definitive. Without direct experimental validation, the precise positioning of many immunodominant epitopes remains uncertain. The experimental epitope characterization performed in this study therefore contributes important empirical evidence to the understanding of NP antigenicity.

SARS-CoV-2 antigen-based assays gained a significant advantage during the pandemic due to their rapid performance and cost-effectiveness. Therefore, anti-NP MAbs have been effectively utilized in developing these tests. Because of NP conserved nature, NP-specific MAbs are more likely to detect the virus across newly identified SARS-CoV-2 variants. In contrast, the spike protein undergoes frequent mutations, leading to significant variations that may negatively affect antigen detection capacity^58^. Throughout the COVID-19 outbreak, NP-specific MAbs were generated as lateral flow immunoassay (LFIA) reagents to robustly detect SARS-CoV-2 NP in nasal swab specimens from patient samples^59^. Dinç et al. also demonstrated that anti-NP MAbs can be employed in both LFIA and electrochemical biosensor technology for clinical SARS-CoV-2 testing^60^. However, some developed tests variably detect different Omicron strains^61^. Therefore, it is essential to ensure that MAbs cross-react with different variants of the virus. This underscores the importance of MAbs that can identify distinct cross-reactive NP epitopes with high affinity and specificity, considering the emergence of new SARS-CoV-2 variants. In this study, high-affinity MAbs with identified epitopes were evaluated as reagents for NP detection in a sandwich ELISA format. A significant advantage of these MAbs is their specificity since they do not cross-react with other hCoVs (NL63, OC43) but specifically recognize the NP of the SARS-CoV-2 and its Omicron variant. Furthermore, iIFA and WB confirmed the ability of the newly generated NP-specific MAbs to detect SARS-CoV-2 NP in infected cells and cell lysates. Altogether, these results highlight the broad applicability of the generated MAbs.

Recent studies demonstrate that SARS-CoV-2 NP can directly bind to the cell surface by interacting with heparan sulfate and heparin, facilitating NP entry into the cell via receptor-mediated endocytosis^34,39^. Notably, compared to NP from other hCoVs, SARS-CoV-2 NP has the highest affinity for cell surface binding^39^. In our study, the performed inhibition assay of NP cellular uptake confirmed that NP_Omircron_ exhibits lung cell binding and enters the cells by endocytosis. Also, it was demonstrated that pretreating recombinant NP with MAbs 4B3, 7F10, 16D9, and 18A8 reduced NP internalization. It is known that SARS-CoV-2 NP induces plenty of cellular responses, including the secretion of proinflammatory cytokines^39^. In that case, inhibiting NP internalization and its interaction with the host’s cellular components may serve as a complementary antiviral strategy, preventing excessive inflammation, reducing viral spread and decreasing COVID-19 severity. For instance, previous studies showed that SARS-CoV-2 NP induces the release of IL-1β and IL-6, thus leading to the activation of NLRP3 inflammasome and subsequent tissue injury^26,32^. Another study demonstrated that NLRP3 inflammasome activation has been observed in COVID-19 patients, correlating with disease severity and clinical outcomes^62^. The sequence spanning aa 260–340 of NP has been identified as a key interaction site with NLRP3, regulating inflammasome activation^32^. Although none of our developed MAbs directly target this region, other studies suggested that blocking this domain could disrupt the initiation of inflammatory processes by inhibiting the assembly and activation of the NLRP3 inflammasome^32^. Beyond its intracellular effects, extracellular NP also contributes to inflammation through complement hyperactivation. Studies have demonstrated that SARS-CoV-2 NP directly binds to MASP-2 protease, a serine protease that plays a key role in the lectin pathway of the complement system, significantly enhancing complement activation^63^. This finding was further supported by Gao and colleagues, who confirmed the interaction between NP and MASP-2. A more detailed analysis of SARS-CoV NP identified its region spanning aa 116–124 as crucial for NP–MASP-2 binding. Given the high sequence similarity between SARS-CoV and SARS-CoV-2, it is likely that blocking this site could prevent MASP-2 association. The identified epitope of 16D9 and 18A8 MAbs (aa 122–136) includes the mentioned NP-MASP-2 interaction site, suggesting that blocking this region may mitigate complement-mediated inflammation^36^.

Taken together, these findings highlight the critical role of SARS-CoV-2 non-surface proteins in viral pathogenesis. As a highly conserved and immunogenic SARS-CoV-2 structural protein, NP serves as a promising target not only for the development of precise diagnostic tools but also for a more in-depth understanding of the molecular mechanisms of virus–host interaction. This study describes novel comprehensively characterized MAbs raised against recombinant NP that are capable to recognize natural SARS-CoV-2 and cross-react with the Omicron variant, which make them promising molecular tools for SARS-CoV-2 detection and research. Moreover, it was found that the epitopes recognized by the MAbs are located in functionally active sites of the NP. This is a crucial characteristic for molecular research as it enables studying protein interactions and biological activities, potentially allowing a direct inhibition or alteration of the protein’s activity. Furthermore, it was demonstrated that several of the developed MAbs (4B3, 7F10, 16D9, 18A8) can inhibit NP internalization in vitro. The observed functional activity of the MAbs has been proven useful for studying virus–host interactions, as the NP is implicated in modulation of the host immune response and viral pathogenesis.

## Materials and methods

### Recombinant proteins

In this study, SARS-CoV-2 full-length recombinant NP and truncated SARS-CoV-2 variant with an N-terminal deletion of aa 1–120 (NP-ΔN) were used for MAb generation. Various recombinant NP constructs, including SARS-CoV-2 Omicron (BA.5) (NP_Omicron_), SARS-CoV NP, NL63 truncated NP with a deletion of N-terminal domain (NL63-NP-ΔN), OC43 NP with a deletion of N-terminal domain (OC43-NP-ΔN) were used in this study for the characterization of MAbs. All recombinant proteins were expressed in *S. cerevisiae* and obtained from UAB Baltymas (Table S1).

### Generation of MAbs against SARS-CoV-2 NP

Two groups of three BALB/c female mice (6–8 weeks old) were immunized subcutaneously with 50 µg of purified SARS-CoV-2 NP or NP-ΔN. Injections were administered every 28 days. For the primary and secondary immunizations, antigens were emulsified in Complete Freund’s adjuvant (Thermo Fisher Scientific) and Incomplete Freund’s adjuvant (Thermo Fisher Scientific), respectively. The third immunization was performed with antigens diluted in phosphate-buffered saline (PBS), pH 7.4. The maintenance of the mice and experimental procedures were performed by certified staff under ARRIVE and FELASA guidelines in accordance with Lithuanian and European legislation. Mice were obtained from the Department of Biological Models (Institute of Biochemistry, Life Sciences Center, Vilnius University), which is licenced by State Food and Veterinary Acency (Vilnius, Lithuania) to breed and use experimental animals for scientific purposes (Vet. Approval No. LT 59–13–001, LT 60–13–001, LT 61–13–004). Permission for immunization experiments was obtained from the Lithuanian State Food and Veterinary Agency (permission No. G2-117, issued 11 June 2019). The immunized mice were sacrificed by cervical dislocation (without anaesthesia) according to the requirements specified in ANNEX IV of DIRECTIVE 2010/63/EU. The death of the mice was confirmed by the onset of *rigor mortis*.

Throughout the immunization process, blood specimens were collected by tail snipping every 28 days and analyzed by indirect enzyme-linked immunosorbent assay (ELISA) to identify the mice with the strongest immune response to the immunogens. Three days before hybridization, selected mice received a booster dose of 50 μg of NP or NP-ΔN in PBS. The hybridization was performed essentially as described by Kohler and Milstein^63,64^. Spleen cells were fused with Sp2/0-Ag14 mouse myeloma cells (CRL-1581™, ATCC) using a polyethylene glycol solution (HybriMax, Sigma-Aldrich) at a ratio of 1:4–1:8. After the fusion, cells were diluted to a final density of 1.25 × 10^6^ cells/mL and cultured in Dulbecco′s Modified Eagle′s Medium (DMEM, Sigma-Aldrich) containing 15% fetal bovine serum (FBS, Sigma-Aldrich) and supplemented with hypoxanthine, aminopterin, and thymidine (50 × HAT media supplement, Sigma-Aldrich) in 96-well cell culture plates (TPP). Viable hybridoma clones producing antigen-specific antibodies were identified using indirect ELISA. Positive clones were stabilized by limiting dilution cloning on a macrophage feeder layer. Stable hybridomas were expanded and cryopreserved for storage. For determining the isotype of the MAbs Mouse Immunoglobulin Isotyping ELISA Kit (550,487, BD Biosciences) was used according to the manufacturer’s instructions.

### Purification of MAbs and conjugation to horseradish peroxidase

MAbs were purified from the hybridoma growth medium by affinity chromatography. Purification was performed on ÄKTA Start (Cytiva) chromatography system using HiTrap Protein A HP column (Cytiva). The column was equilibrated with 10 column volumes (CV) of washing buffer (1.5 M glycine, 3 M NaCl, pH 8.9), and hybridoma supernatant diluted with washing buffer at a 1:2 ratio was loaded. After washing with 20 CV of washing buffer, bound antibodies were eluted using elution buffer (0.1 M glycine, pH 3.0). The MAbs were dialyzed against PBS overnight (ON) at 4 °C, sterile-filtered, and stored at 4 °C.

Purified MAbs were conjugated with horseradish peroxidase (HRP) using the periodate method, as previously described^65^. The dilution of conjugates for further experiments was selected from the titration curve corresponding to the optical density (OD) value of approximately 2 at 450 nm.

#### ELISA

An indirect Enzyme-Linked ImmunoSorbent Assay (ELISA) was used to analyze blood samples from the immunized mice, select positive hybridoma clones, and characterize the specificity and affinity of the purified MAbs. The 96-well polystyrene plates (MaxiSorp, Fisher Scientific) were coated with either SARS-CoV-2 NP, NP-ΔN, NP_Omicron_ protein diluted in coating buffer (0.05 M sodium carbonate, pH 9.5) to a concentration of 3 µg/mL and incubated ON at 4 °C. The wells were blocked with 2% bovine serum albumin (BSA) solution in PBS for 1 h at room temperature (RT). Mice blood samples (starting dilution 1:200), purified MAbs (3 µg/mL) were serially diluted in PBS-T buffer (0.1% Tween-20 in PBS), or undiluted hybridoma supernatants were added to the wells, followed by 1 h incubation at RT. After washing the plates 5 times with PBS-T, Goat Anti-Mouse IgG (H + L)-HRP (1721011, Bio-Rad) was added at a dilution of 1:5000 in PBS-T and incubated for 1 h RT. The plates were washed as before. The enzymatic reaction was developed using 3,3’,5,5’-Tetramethylbenzidine (TMB) substrate (01016-1, Clinical Science Products) for 7–10 min at RT and stopped by adding 3.6% sulfuric acid. The OD was measured by plate spectrophotometer (Multiskan GO, Thermo Fisher Scientific) as a difference of OD between 450 and 620 nm (reference wavelength). Each MAb’s apparent dissociation constant (K_d_) values were calculated from the ELISA titration curves and defined as a picomolar (pM) concentration of MAb corresponding to the mid-point between the maximum OD450 value and the background.

The activity of MAb-HRP conjugates was tested by a direct ELISA. The conjugates were serially diluted in PBS-T (starting from 1:100) and added to the polystyrene plates coated with NP. The blocking, washing and signal detection steps were performed as above.

A competitive ELISA was used to determine if the MAbs compete for a binding site. The NP immobilization and blocking were done as in an indirect ELISA. The coated antigen was incubated with 40 µg/mL of purified MAbs for 1 h at RT. MAb-HRP conjugates were added to the wells (dilutions according to the results of direct ELISA) and incubated further for 1 h at RT. The plates were washed, and a signal was detected. The competition of MAbs for the recognized epitopes was defined from the OD values in accordance with positive (MAb competing with its HRP conjugate) and negative (direct MAb-HRP interaction with antigen without competing MAb) controls.

A sandwich ELISA was performed to detect NP using a pair of different epitope-recognizing capture and detection MAbs. Several parameters were evaluated for system optimization, including concentration and buffer conditions for MAb 6G11 immobilization, blocking conditions, and the dilution of MAb 12B2-HRP. Capture MAbs diluted in PBS to a final concentration of 3 µg/mL were immobilized in polystyrene plates and incubated ON at 4 °C. The blocking was done using 1 × Roti-Block (Roth) solution for 1 h at RT. After adding NP (starting concentration 1 µg/mL), plates were incubated for 1 h RT, then washed, and HRP-labeled MAbs were added for 1h RT. The enzymatic reaction was initiated and stopped as described above.

### Antibody binding kinetics analysis

Bio-layer interferometry (BLI) was used for antibody binding to SARS-CoV-2 NP_Omicron_ kinetics measurements on Octet K2 (ForteBio) instrument. The binding assays were performed in 96-well black plates (Greiner) at 30 °C with 1000 rpm orbital shake speed. All samples were diluted in freshly prepared and filtered PBS with 0.1% (w/v) Tween-20 and 5 mg/ml BSA, and 220 µL was added per well. The MAbs were immobilized using anti-mouse IgG Fc Octet AMC biosensors (Sartorius). The biosensors were preincubated for at least 10 min in the assay buffer prior to the measurement start. The data acquisition was performed during these steps: baseline incubation for 30 s, antibody loading for 300 s, baseline for 30 s, antigen association for 600 s, and dissociation for 800 s. Except for MAbs 16D9 and 18A8, association was measured for 180 s, and dissociation for 360 s. The exact MAbs and NP_Omicron_ concentrations used are indicated in the supplementary Fig. S1a. In every assay, a biosensor loaded with the MAb was dipped in a well containing only assay buffer, and the signal measured was used for reference sample subtraction, except for MAbs 16D9 and 18A8, for which reference sensor measurements were also used for subtraction. The binding sensorgrams were collected using 2-channel mode on Octet K2, and only fresh biosensors were used for each measurement. The data was analysed using Octet Analysis Studio 12.2.2.26 software (Sartorius). For the binding curves data, single reference sample subtraction, alignment to the average of the baseline step, and global fit to a 1:1 binding model were performed. The results are reported as value ± standard error of the mean (SEM).

### Preparation of SARS-CoV-2 infected cell lysates

TMPRRS2 receptor expressing Vero E6 cells (kindly gifted by V. Thiel, University of Bern, Switzerland) were seeded into 6-well cell culture plates (Thermo Scientific) for ON incubation prior to virus infection. Two different SARS-CoV-2 strains were used in the study: wild-type (WT) SARS-CoV-2 (SARS-CoV-2 Germany/BavPat1/2020 (BavPat1) lineage B.1; GISAID accession EPI_ISL_406862) and Omicron XBB.1.5 variant (hCoV-19/Netherlands/NH-EMC-5667/2023; GISAID accession EPI_ISL_16640568). The cells were incubated with viruses for 48 h at 37 °C. After the incubation, the medium was discarded, and 500 µL of cold lysis buffer was added (0.02 M Na_2_HPO_4_, 0.002 M EDTA, 0.15 M NaCl, 1% Triton X-100, pH 7.6), followed by an incubation for 1 h at 4 °C. Later, the cells were centrifuged, and the supernatant was mixed with 100 µL of PBS and 100 µL SDS-PAGE buffer. The preparation of lysates was performed in a biosafety level 3 laboratory.

### Western Blot

Western blot (WB) analysis was performed as described previously^66^. Briefly, recombinant protein samples (0.5 µg/lane), *S. cerevisiae* (AH22–214 strain kindly gifted by M. Norkienė, Vilnius University, Lithuania) or *E. coli* lysates (2–7 µL/lane), and Vero E6 cell lysates (15 µL/lane) were fractioned in sodium dodecyl sulfate–polyacrylamide gel electrophoresis (SDS-PAGE) using 12% polyacrylamide gels under denaturing and reducing conditions. Proteins were transferred to a polyvinylidene fluoride (88518, Thermo Scientific) or nitrocellulose (10600001, Cytiva) membrane, followed by blocking the membrane with 2–5% milk powder in PBS for 1 h at RT. The membranes were incubated for 1 h at RT or ON at 4 °C with purified MAbs diluted to 0.5–5 µg/mL in PBS-T containing 2–5% milk powder. After washing with PBS-T, the membranes were incubated for 1 h with Goat Anti-Mouse IgG (H + L)-HRP (1721011, Bio-Rad) diluted 1:4000 or Peroxidase-conjugated AffiniPure™ F(ab’)2 Fragment Goat Anti-Mouse IgG (H + L) (115-036-146, Jackson ImmunoResearch) diluted 1:10,000 in PBS-T containing 2–5% milk powder. Following additional washing, the bands of proteins were visualized colorimetrically using 1-Step™ TMB-Blotting Substrate Solution (34018, Thermo Scientific) or by chemiluminescence using 900 µL of each Clarity™ Western ECL Substrate luminol and peroxide solution (1705061, Bio-Rad), incubating for 5 min.

### Microarray ELISA

This approach was used to analyze naturally occurring and MAbs-recognized epitopes due to its low serum sample volume requirement, making it suitable for limited specimens. Blood serum samples confirmed as SARS-CoV-2 anti-NP IgG positive by Coronavirus IgG MaE Test (UAB Imunodiagnostika, Lithuania) and serum samples confirmed as negative using this test were used for microarray ELISA (MaE)^67^. Informed written consent was obtained from all subjects involved. The study was approved by Vilnius Regional Biomedical Research Ethics Committee (approval No:0.2021/10˗1385˗857). All experiments concerning human ethics were performed in accordance with the Declaration of Helsinki.

Microarrays in transparent 96-well plates (3D-Epoxy, PolyAn) were printed using sciFLEXARRAYER SX (Scienion). Recombinant SARS-CoV-2 NP and NP_Omicron_ protein samples were diluted in 0.05 M sodium carbonate buffer, pH 9.5, to the concentrations of 0.88 and 0.36 mg/mL, respectively. For control spots, human IgG (0150–01, SouthernBiotech) solution in PBS at the concentration of 0.4 mg/mL was used. Each spot in the microarray is formed by dispensing 1 drop (450 pL) of the protein sample. After printing, the plates were incubated for 16 h at 2–8 °C, dried for 15 min at 37 °C, and blocked using 300 µL per well of PBS-T with 2% (m/v) BSA for 1 h at RT. After blocking, the wells were emptied and dried for 1 h at 37 °C, followed by packing with a desiccant bag in vacuum-sealed bags for storage at 2–8 °C until used. Each MAb, at a concentration of 15 µg/mL in blocking buffer, was added to the wells and incubated for 2 h at RT (15 positive and 3 negative) were diluted 1:500 in the blocking buffer and incubated with each MAb for 1 h at RT. Following incubation, the plates were rinsed with PBS-T, then secondary Mouse Anti-Human IgG-Fc antibodies conjugated with HRP diluted 1:4000 in blocking buffer (9040–05, SouthernBiotech) were added and incubated for 1 h at RT. After the plates were rinsed, a precipitating TMB substrate (T0565, Sigma-Aldrich) was used to develop the signal. The enzymatic reaction was stopped by washing the plates with water. The image acquisition and analysis were performed using sciREADER CL2 (Scienion) and its software.

### Epitope mapping

The mapping of epitopes recognized by the developed MAbs was performed by WB using a set of SARS-CoV-2 NP fragments. Six overlapping DNA fragments spanning the full-length NP sequence (P0DTC9) were amplified by PCR (Table S2, fragments 1–6). Amplified fragments were purified using GeneJET Gel Extraction Kit (Thermo Fisher Scientific) and cloned into pJET1.2 vector using CloneJET PCR Cloning Kit (Thermo Fisher Scientific) according to the manufacturer’s recommendations. To produce N-terminal His-tagged protein fragments, the inserts were digested with a pair of NheI and BamHI or NheI and XhoI (Thermo Fisher Scientific) restriction endonucleases and ligated into pET28a(+) (Thermo Fisher Scientific) vector. The recombinant plasmids were verified by sequencing and transformed into *E. coli* Rosetta-gami 2(DE3) (Novagen, Merck) strain. The protein expression was induced with 0.001 M isopropyl β-D-1-thiogalactopyranoside (IPTG, Thermo Fisher Scientific); after 5 h of cultivation at 37 °C, cells were collected. The reactivity of the MAbs with the NP fragments was analyzed by WB.

For detailed epitope mapping of the MAbs, 13 truncated fragments were generated and expressed in *E. coli* Tuner(DE3) (Novagen, Merck) strain (Table S2, fragments 7–19). The fragments were amplified and cloned into pJET1.2 vector as described previously. The inserts were hydrolyzed with a pair of BamHI and XhoI or KpnI and XhoI restriction endonucleases and ligated into pET43.1a(+) (Novagen, Merck) vector to produce N-terminal His-tagged protein fragments. The protein expression was induced with 0.001 M IPTG; after 2.5 h of cultivation at 37 °C, cells were collected. The synthesis of fragments and the reactivity of the MAbs were analyzed as described above.

In silico computational analysis of epitopes was performed using MAFFT software for sequence alignment, and the results were visualized with Jalview. The sequences of NP of NL63, OC43, SARS-CoV, SARS-CoV-2 WT, and SARS-CoV-2 Omicron variants BA.5 and JN.1 were taken from UniProt or NCBI GenBank database (accession numbers Q6Q1R8, P33469, P59595, P0DTC9, UOZ45812.1, XLG88095.1, respectively).

### Immunofluorescence assay

An indirect immunofluorescence (iIFA) assay for MAb staining of SARS-CoV-2-infected cells was performed as described previously^67^. In brief, Vero E6 cells (CRL-1586 ™, ATCC) were seeded into a 96-well cell culture plate and infected with the 2019 nCoV BavPAt1 strain of SARS-CoV-2 WT virus. After 24 h, cells were fixed with 4% paraformaldehyde and permeabilized with 0.5% Triton X-100. A concentration of 10 µg/mL of purified MAbs was used for the analysis. A secondary Goat Anti-Mouse FITC-labeled antibody (F047902-2, Agilent Technologies) (diluted 1:200) was added for detection. The cells were analyzed by fluorescence microscopy using a Nikon Eclipse Ti inverted fluorescence microscope. Images were prepared with ImageJ program with brightness and contrast adjusted equally to all images per channel.

iIFA was also used for the internalization of NP_Omicron_ in cells. The cells were fixed and permeabilized, as described above. A concentration of 10 µg/mL of purified MAb 6G11 was used for the analysis. Goat anti-Mouse IgG (H + L) Superclonal™ Secondary Antibody, labeled with Alexa Fluor™ 488 (A28175, Thermo Scientific) (diluted 1:2000) was used for detection. The cells were analyzed by Olympus IX-81 fluorescence microscope. Images were prepared with ImageJ program, with brightness and contrast adjusted equally to all images per channel.

### MAb-mediated inhibition of SARS-CoV-2 NP cellular uptake

For the MAb-mediated inhibition assay, IMR-90 cells (CCL-186™, ATCC) were seeded into a 384-well plate (Corning) at a density of 7 × 10^4^ cells/mL in Eagle’s Minimum Essential Medium (EMEM, ATCC) containing 10% FBS and incubated for 24 h at 37 °C for optimal confluency. NP_Omicron_ was labeled with pHrodo™ Deep Red TFP Ester (P35358, Thermo Scientific) according to the manufacturer’s protocol. The final amount of 2 µg of labeled NP_Omicron_ (NP_Omicron_-Deep Red) was preincubated with 15 µg of each MAb specific to SARS-CoV-2 NP for 1 h at RT. As an isotype control in house-made MAb 5E2 (IgG1 subtype, κ light chain) against β-lactamase NDM-1 was used (previously generated at Vilnius University, Life Sciences Center). The protein-antibody complexes were added to the cells and incubated for 4 h at 37 °C. To inhibit endocytosis, the cells were pretreated with 30 µM chlorpromazine hydrochloride (CPZ) and incubated at 37 °C for 30 min. After staining, Hoechst 33342 trihydrochloride (H3570, Thermo Scientific) was used at a concentration of 5 µg/mL for nucleus staining, while LysoBrite™ Green (22643, AAT Bioquest) was used for lysosome staining, following the manufacturer’s recommended concentration. The cells were analyzed by fluorescence microscopy using live cell imaging chamber supplied by 5% CO_2_ at 37 °C. The preparation of images is described above.

### Data analysis

The apparent K_d_ values were calculated using OriginPro 8 software (v8.0951, https://www.originlab.com/), and the results were presented as mean ± standard error of the mean (SEM), n = 3. The data of antibody binding kinetics was analyzed using Octet Analysis Studio 12.2.2.26 software (Sartorius). For the binding curves data, single reference sample subtraction, alignment to the average of the baseline step, and global fit to a 1:1 binding model were performed. The results are reported as value ± SEM. WB band intensities were quantified using ImageJ software (v1.54d, https://imagej.net/ij/) by converting images to 8-bit grayscale and analyzing peaks. The area under each peak was used to determine relative signal intensity. Binding affinities of the MAbs were visualized as heatmaps in Microsoft Excel (v2507, https://www.microsoft.com/) using a three-color scale, with lowest, 50th percentile, and highest values set to the minimum, midpoint, and maximum signals, respectively. ELISA and BLI data were analyzed separately. The standard curve parameters (R^2^) and the limit of detection for the optimized sandwich ELISA were determined using the same software and according to^37^, respectively. Graphs were prepared using GraphPad Prism software (v9.5.1, https://www.graphpad.com/). Mean fluorescence intensity (MFI) calculations (n = 3) were done using Image J (v1.54d, https://imagej.net/ij/) For MFI data, comparisons were made using the Student’s t-test in GraphPad Prism. All data are shown as the mean ± SEM with *p* < 0.05 considered statistically significant.

## Supplementary Information

Below is the link to the electronic supplementary material.

Supplementary Material 1

## Data Availability

The datasets generated and analyzed during the current study are provided within the manuscript and supplementary information files. Any additional raw data are available from the corresponding author (Agnė Rimkutė; agne.rimkute@gmc.vu.lt) upon reasonable request.

## Abbreviations

BLI: Bio-layer interferometry
BSA: Bovine serum albumin
CPZ: Chlorpromazine hydrochloride
CTD: C-terminal domain
ELISA: Enzyme-linked immunosorbent assay
HCoVs: Human coronaviruses
HRP: Horseradish peroxidase
IFN: Interferon
iIFA: Indirect immunofluorescence assay
K_d_: Dissociation constant
LFIA: Lateral flow immunoassay
LKR: Central linker region
MAbs: Monoclonal antibodies
MaE: Microarray ELISA
MFI: Mean fluorescence intensity
NP: SARS-CoV-2 nucleocapsid protein
NTD: N-terminal domain
OD: Optical density
ON: Overnight
RT: Room temperature
SEM: Standard error of the mean
TMB: 3,3’,5,5’-Tetramethylbenzidine
WB: Western blot
WT: Wild type
