# Supplementary material for: SARS-CoV-2 nucleocapsid protein-specific monoclonal antibodies as tools for studying its antigenic structure and interaction with host cells
Source: Sci Rep. 2026 Feb 28;16:11461. doi: 10.1038/s41598-026-40984-8 (PMC13057025; doi:10.1038/s41598-026-40984-8)
Supplement: Supplementary file 1 — Supplementary Material 1 [file 41598_2026_40984_MOESM1_ESM.pdf]

SUPPLEMENT

TABLES

Table S1. The Uniprot and NCBI GenBank accession numbers of NPs of SARS-CoV-2 and other hCoVs used in the study.

Protein	Cat. No.	aa	Accession number
NP	20-S2N-ScB-F	419	P0DTC9
NP-ΔN	21-S2delNN-ScB-F	299	P0DTC9
$NP_{Omicron}$	22-S2NOM5-ScB-F	416	UOZ45812.1
SCoV-NP	20-S1N-ScB-F	422	P59595
NL63-NP-ΔN	21-NL63delNN-ScB-F	377	Q6Q1R8
OC43-NP-ΔN	21-OC43delNN-ScB-F	448	P33469

Table S2. PCR primers used for generating fragments of SARS-CoV-2 NP.

Fragment No.	Fragment length		Primer sequences 5'-3'	Restriction endonucleases
	bp	aa		
N1	1-600	1-200	GCAGGCTAGCATGTCTGATAATGGTCCAC	NheI BamHI or NheI XhoI
			GCAGGATCCTTAACCTGGAGTAGAGTTTCTAGA	
N2	280-600	94-200	GCAGGCTATTAGGGGTGGTGATGG	
			GCAGGATCCTTAACCTGGAGTAGAGTTTCTAGA	
N3	520-1026	173-342	GCAGGCTAGCGAAGGTTCTAGAGGTGGTT	
			GCACTCGAGTTATTCTGTTGGTGGGAATGTTT	
N4	520-1101	173-367	GCAGGCTAGCGAAGGTTCTAGAGGTGGTT	
			GCACTCGAGTTATTCTGTTGGTGGGAATGTTT	
N5	682-1026	228-342	GCAGGCTAGCAACCAACTGGAATCTAAAATG	
			GCAGGATCCTTAAGCTTGTGTAGAATCA	
N6	682-1260	228-419	GCAGGCTAGCAACCAACTGGAATCTAAAATG	
			GCAGGATCCTTAAGCTTGTGTAGAATCA	
N1.1	1-279	1-93	ATAGGATCCATGTCTGATAATGGTCCAC	BamHI XhoI
			ATACTCGAGTTAACCACCCCTAATTCTTCTT	
N1.2	52-279	18-93	ATAGGATCCGGTGGTCCATCTGATTCTAC	
			ATACTCGAGTTAACCACCCCTAATTCTTCTT	
N1.3	109-279	37-93	ATAGGATCCACTAAACAAAGAAGACCACA	
			ATACTCGAGTTAACCACCCCTAATTCTTCTT	
N1.4	163-279	55-93	ATAGGATCCGCTCTAACACAACATGGTA	
			ATACTCGAGTTAACCACCCCTAATTCTTCTT	
N1.5	202-279	68-93	ATAGGATCCAGAGGTCAAGGTGTTCCAAT	
			ATACTCGAGTTAACCACCCCTAATTCTTCTT	
N2.1	361-519	121-172	ATAGGATCCCCATATGGTGCTAATAAAGATG	
			ATCCTCGAGTTAATCAGCATAGAATCCTTTTGG	
N2.2	409-519	137-172	ATAGGATCCACAGGTGCTCTAAATACACCAAA	
			ATCCTCGAGTTAATCAGCATAGAATCCTTTTGG	
N2.3	463-519	155-172	ATAGGATCCCTAAGAAATCCAGCTAATAATGCT	
			ATCCTCGAGTTAATCAGCATAGAATCCTTTTGG	
N3.1	652-681	218-227	ATAGGATCCAAAATGGCTGGTAATGGTG	
			ATACTCGAGTAATCCAGTTGGTTTAATCTATC	
N6.1	1027-1260	343-419	ATAGGTACCAAGATCCAAACTTCAAAGATCAA	KpnI XhoI
			ATACTCGAGCTTAAGCTTGTGTAGAATCAGCAG	
N6.2	1075-1260	359-419	ATAGGTACCAAGCTTATAAAACATTCCCACCA	
			ATACTCGAGCTTAAGCTTGTGTAGAATCAGCAG	
N6.3	1126-1260	376-419	ATAGGTACCAAGCCGATGAGACACAAGCT	
			ATACTCGAGCTTAAGCTTGTGTAGAATCAGCAG	
N6.4	1186-1260	396-419	ATAGGTACCAACCAGCTGCTGATCTAGATG	
			ATACTCGAGCTTAAGCTTGTGTAGAATCAGCAG	

Table S3. Summary table of epitope mapping by Western Blot using recombinant SARS-CoV-2 NP fragments. "+" indicates MAb binding to a fragment; "-" indicates no binding; grey-colored box represents binding not tested.

Fragments	aa	MAbs								
		1A6	4B3	4G6	6G11	7F10	12B2	13C10	16D9	18A8
N1	1-200	-	-	-	-	+	-	-	-	-
N2	94-200	-	-	-	-	-	-	-	+	+
N3	173-342	-	-	-	-	-	-	+	-	-
N4	173-367	+	-	+	+	-	+	+	-	-
N5	228-342	-	-	-	-	-	-	-	-	-
N6	228-419	+	+	+	+	-	+	-	-	-
N1.1	1-93					-				
N1.2	18-93					-				
N1.3	37-93					-				
N1.4	55-93					-				
N1.5	68-93					-				
N2.1	121-172								+	+
N2.2	137-172								-	-
N2.3	155-172								-	-
N3.1	218-227							+		
N4.1	343-419	-	+	-	-		-			
N4.3	359-419	-	-	-	-		-			
N4.3	376-419	-	-	-	-		-			
N4.4	396-419	-	-	-	-		-			

Table S4. SARS-CoV-2 NP amino acid changes in past variants of concern and current variants of interest or under monitoring, as classified by WHO (https://www.who.int/activities/tracking-SARS-CoV-2-variants). NP variants used for MAb epitope prediction analysis are bolded.

Variant	Amino Acid Changes in NP										
Alpha	D3L						R203K	G204R		S235F	
Beta									T205I		
Gamma					P80R		R203K	G204R			
Delta				D63G			R203M		G215C	D377Y	
BA.1		P13L	Δ31-33				R203K	G204R			
BA.2		P13L	Δ31-33				R203K	G204R			S413R
BA.4		P13L	Δ31-33			P151S	R203K	G204R			S413R
**BA.5**		**P13L**	**Δ31-33**				**R203K**	**G204R**			**S413R**
**XBB.1.5**		P13L	Δ31-33				R203K	G204R			S413R
XEC		P13L	Δ31-33				R203K	G204P	Q229K		S413R
**JN.1**		**P13L**	**Δ31-33**				**R203K**	**G204R**	**Q229K**		**S413R**

Table S5. Microarray ELISA results demonstrating no inhibition of human anti-SARS-CoV-2 NP IgG binding to the antigen in presence of SARS-CoV-2 NP specific MAbs. As a negative control, the sera of SARS-CoV-2 anti-NP IgG-negative individuals were used (negative #1, negative #2, negative #3). The values represent the median intensity of the corresponding microarray spots in relative units

		**Serum sample**											
		**#1**		**#2**		**#3**		**#4**		**#5**		**#6**	
		**NP**	**NP$_{Omicron}$**	**NP**	**NP$_{Omicron}$**	**NP**	**NP$_{Omicron}$**	**NP**	**NP$_{Omicron}$**	**NP**	**NP$_{Omicron}$**	**NP**	**NP$_{Omicron}$**
**MAb**	1A6	10.1	17.9	15.4	10.7	17.7	13.0	13.5	8.6	19.4	13.2	2.9	1.1
	4B3	11.4	20.9	25.9	7.5	24.3	6.1	25.1	7.5	13.7	13.9	1.5	3.4
	4G6	7.1	11.4	19.7	8.2	22.9	7.0	22.3	8.0	12.9	4.1	0.6	0.9
	6G11	9.4	4.8	18.1	3.9	19.0	2.2	15.7	7.2	20.5	15.4	1.5	1.0
	7F10	12.7	11.8	19.8	5.6	24.9	5.5	22.7	12.5	11.6	12.1	6.2	3.1
	12B2	5.1	7.7	22.2	4.1	24.7	3.4	22.4	6.1	14.2	22.7	3.1	4.5
	13C10	7.8	10.9	20.2	5.8	22.9	5.1	24.0	8.1	20.2	9.4	5.1	6.2
	**No MAb**	**14.5**	**8.2**	**29.0**	**14.1**	**20.3**	**10.6**	**16.5**	**14.9**	**18.4**	**14.2**	**14.9**	**17.6**
		**#7**		**#8**		**#9**		**#10**		**#11**		**#12**	
		**NP**	**NP$_{Omicron}$**	**NP**	**NP$_{Omicron}$**	**NP**	**NP$_{Omicron}$**	**NP**	**NP$_{Omicron}$**	**NP**	**NP$_{Omicron}$**	**NP**	**NP$_{Omicron}$**
**MAb**	1A6	12.6	1.3	7.9	1.4	16.7	1.6	6.3	0.1	6.2	0.5	14.8	1.2
	4B3	13.0	1.6	10.6	3.0	11.7	12.9	6.5	0.1	7.4	0.2	19.1	2.1
	4G6	12.8	3.0	8.6	5.5	7.6	7.9	5.0	0.1	5.6	0.7	10.5	1.7
	6G11	14.7	2.1	12.7	1.2	4.9	7.3	6.5	0.3	5.2	0.2	12.3	1.8
	7F10	10.9	0.8	8.2	0.5	15.2	6.6	6.7	5.0	3.3	0.6	12.9	5.0
	12B2	13.0	2.2	11.1	2.8	7.7	9.7	8.5	0.3	7.5	0.4	14.9	1.3
	13C10	13.2	2.0	11.5	1.3	9.0	9.3	9.8	1.8	9.3	1.7	17.4	1.5
	**No MAb**	**13.0**	**1.5**	**15.0**	**4.2**	**6.4**	**7.3**	**11.0**	**0.4**	**8.7**	**1.9**	**24.0**	**2.3**
		**#13**		**#14**		**#15**		**Negative #1**		**Negative #2**		**Negative #3**	
		**NP**	**NP$_{Omicron}$**	**NP**	**NP$_{Omicron}$**	**NP**	**NP$_{Omicron}$**	**NP**	**NP$_{Omicron}$**	**NP**	**NP$_{Omicron}$**	**NP**	**NP$_{Omicron}$**
**MAb**	1A6	6.2	0.5	6.7	0.1	0.1	0.0	0.4	2.2	0.4	3.6	0.2	0.2
	4B3	7.4	0.2	6.2	0.2	2.0	0.3	0.4	0.1	1.6	0.1	0.4	0.1
	4G6	5.6	0.7	7.1	0.3	1.5	0.0	0.2	0.3	0.1	0	0.0	0.3
	6G11	5.2	0.2	8.4	0.1	0.1	0.0	1.8	8.5	0.2	0.3	0.0	0.0
	7F10	3.3	0.6	1.2	0.2	1.5	0.1	0.1	0.2	0.1	0.2	0.0	1.9
	12B2	7.5	0.4	4.6	0.1	5.7	0.0	0.1	0.3	0.1	0.6	0.2	0.0
	13C10	9.3	1.7	5.8	0.6	4.9	1.5	0.1	0.1	0.6	0.2	0.0	0.2
	**No MAb**	**8.7**	**1.9**	**11.3**	**0.3**	**5.5**	**0.9**	**0.1**	**0.1**	**0.1**	**0.9**	**0.3**	**0.1**

FIGURES

a

MAb	MAb Conc. (μg/mL)	Load. Response (nm)	$NP_{Omicron}$ Conc. (nM)	Bind. Response (nm)	K_D (M)	K_D Error	k_a ($M^{-1}s^{-1}$)	k_a Error	k_d (s^{-1})	k_d Error	Rmax	Rmax Error	Full X^2	Full R^2
		0.90	25	0.94							0.98	0.00		
4G6	5	0.94	12.5	0.94	1.53×10^{-9}	1.09×10^{-11}	1.91×10^{5}	8.83×10^{2}	2.91×10^{-4}	1.59×10^{-6}	1.25	0.00	40.3	0.98
		0.93	6.25	0.72							1.50	0.01		
		0.93	3.13	0.41							1.57	0.01		
		0.86	12.5	0.67							0.74	0.00		
7F10	2	0.86	6.25	0.56	7.74×10^{-10}	8.14×10^{-12}	2.93×10^{5}	1.75×10^{3}	2.27×10^{-4}	1.96×10^{-6}	0.85	0.00	16.2	0.97
		0.89	3.13	0.32							0.79	0.00		
		0.97	25	0.92							1.32	0.01		
1A6	2	0.89	12.5	0.84	3.28×10^{-9}	3.80×10^{-11}	7.90×10^{4}	7.22×10^{2}	2.59×10^{-4}	1.84×10^{-6}	1.88	0.01	28.2	0.98
		0.93	6.25	0.50							2.13	0.02		
		0.67	12.5	0.52							0.53	0.00		
4B3	5	0.67	6.25	0.43	1.09×10^{-9}	1.01×10^{-11}	3.91×10^{5}	2.68×10^{3}	4.25×10^{-4}	2.70×10^{-6}	0.58	0.00	15.9	0.95
		0.70	3.13	0.27							0.60	0.00		
		0.80	12.5	0.80							0.87	0.00		
6G11	2	0.79	6.25	0.61	7.04×10^{-10}	9.51×10^{-12}	2.84×10^{5}	2.01×10^{3}	1.99×10^{-4}	2.30×10^{-6}	0.95	0.01	29.5	0.97
		0.80	3.13	0.35							0.90	0.01		
		0.89	12.5	0.74							0.85	0.00		
12B2	5	0.91	6.25	0.62	9.62×10^{-10}	1.26×10^{-11}	2.45×10^{5}	2.01×10^{3}	2.36×10^{-4}	2.40×10^{-6}	1.05	0.01	28.4	0.97
		0.91	3.13	0.32							0.94	0.01		
		0.86	50	0.88							1.88	0.02		
13C10	2	0.83	25	0.70	7.27×10^{-9}	1.18×10^{-10}	2.44×10^{4}	3.15×10^{2}	1.77×10^{-4}	1.76×10^{-6}	2.01	0.02	34.4	0.98
		0.81	12.5	0.57							3.07	0.04		
		0.80	6.25	0.26							2.82	0.04		
		0.84	1000	-0.02										
		0.84	333.3	-0.02										
16D9	2	0.95	111.1	-0.01										
		0.93	37	-0.01										
		0.94	12.4	0.01										
		0.95	1000	0.03										
18A8	2	0.94	333.3	0.02										
		0.98	111.1	0.01										

b

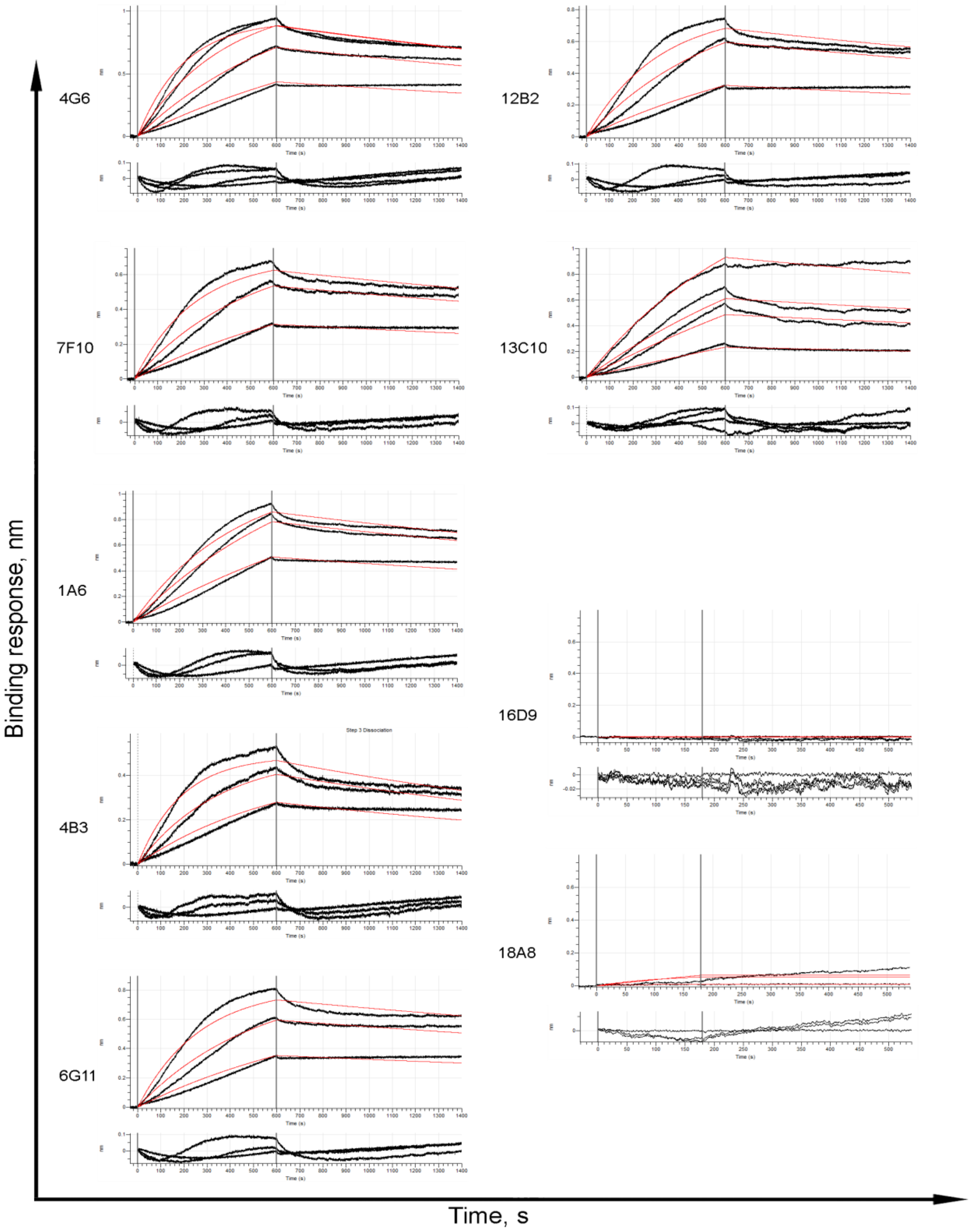

Fig. S1. Binding kinetics of the MAbs to $NP_{Omicron}$ analysis in bio-layer interferometry. Information table on the ligand and analyte concentrations used and the parameters calculated (a); Sensorgrams of all the measurements. Antibody used is indicated on the left, the above graph is the binding response curve, and the below is a residuals plot of the 1:1 model fit (b).

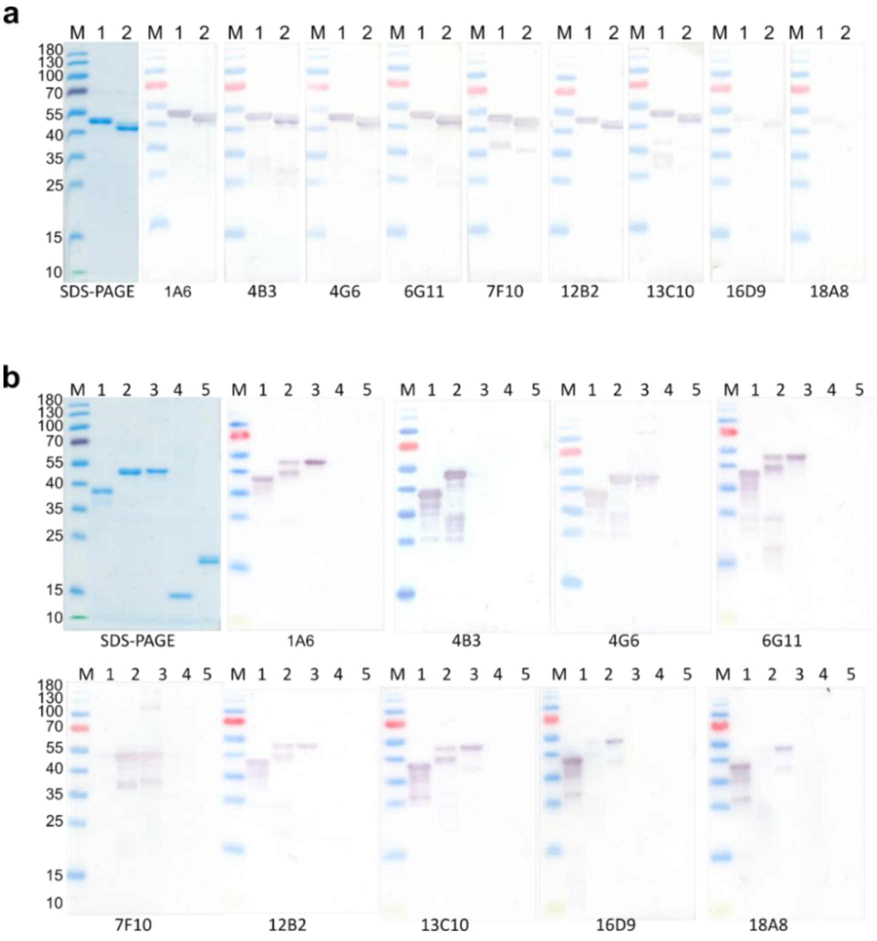

Fig S2. Western Blot analysis of anti-NP MAbs reactivity with recombinant SARS-CoV-2 NPs: SARS-CoV-2 full-length NP (NP) (lane 1) and SARS-CoV-2 NP Omicron (BA.5) variant ($NP_{Omicron}$) (lane 2) (a); truncated SARS-CoV-2 NP (NP-ΔN) (lane 1), NP (lane 2), SARS-CoV NP (lane 3), OC43 NP (OC43-NP-ΔN) (lane 4), and NL63 NP (NL63-NP-ΔN) (lane 5) (b). M represents protein molecular weight standard (PageRuler™ Prestained Protein Ladder, 10 to 180 kDa, Thermo Scientific).

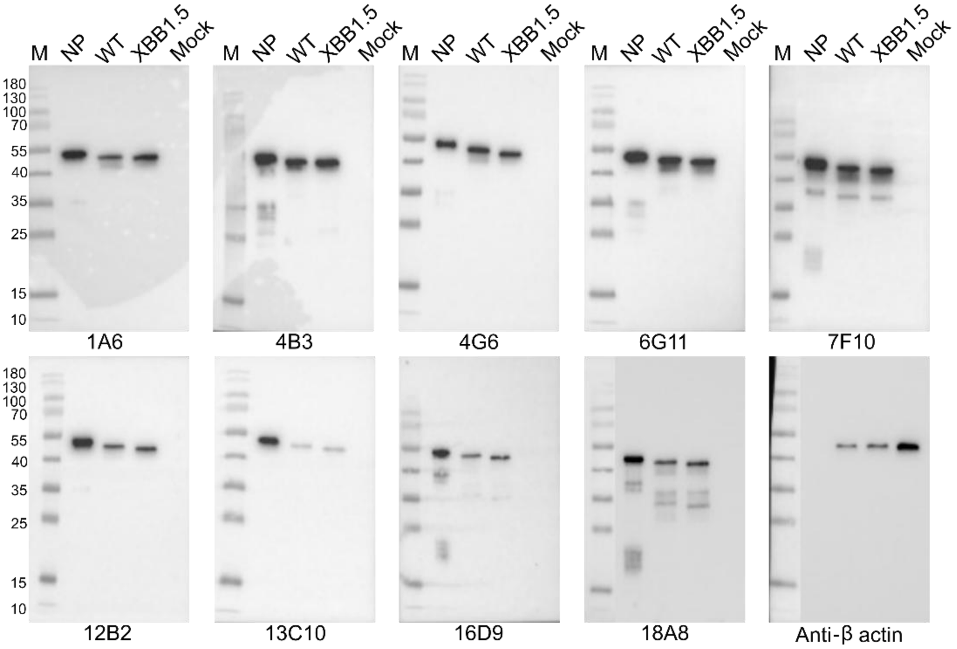

Fig. S3. Original Western Blot analysis of SARS-CoV-2 NP specific MAbs reactivity with SARS-CoV-2 wild-type virus (SARS-CoV-2 Germany/BavPat1/2020) and Omicron XBB1.5 variant (hCoV-19/Netherlands/NH-EMC-5667/2023) infected Vero E6 cell lysates. Mock indicates non-infected cells lysate. M represents protein molecular weight standard (PageRuler™ Prestained Protein Ladder, 10 to 180 kDa, Thermo Scientific).

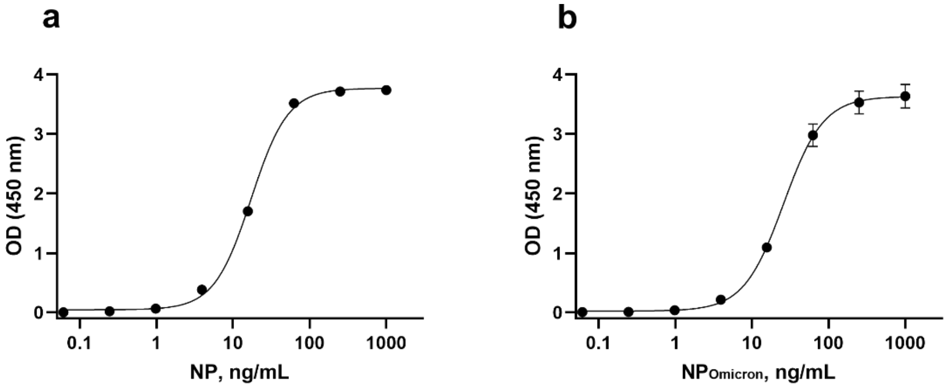

Fig S4. The standard curves of optimized sandwich ELISA for NP (a) and $NP_{Omicron}$ (b) quantification (n = 3).

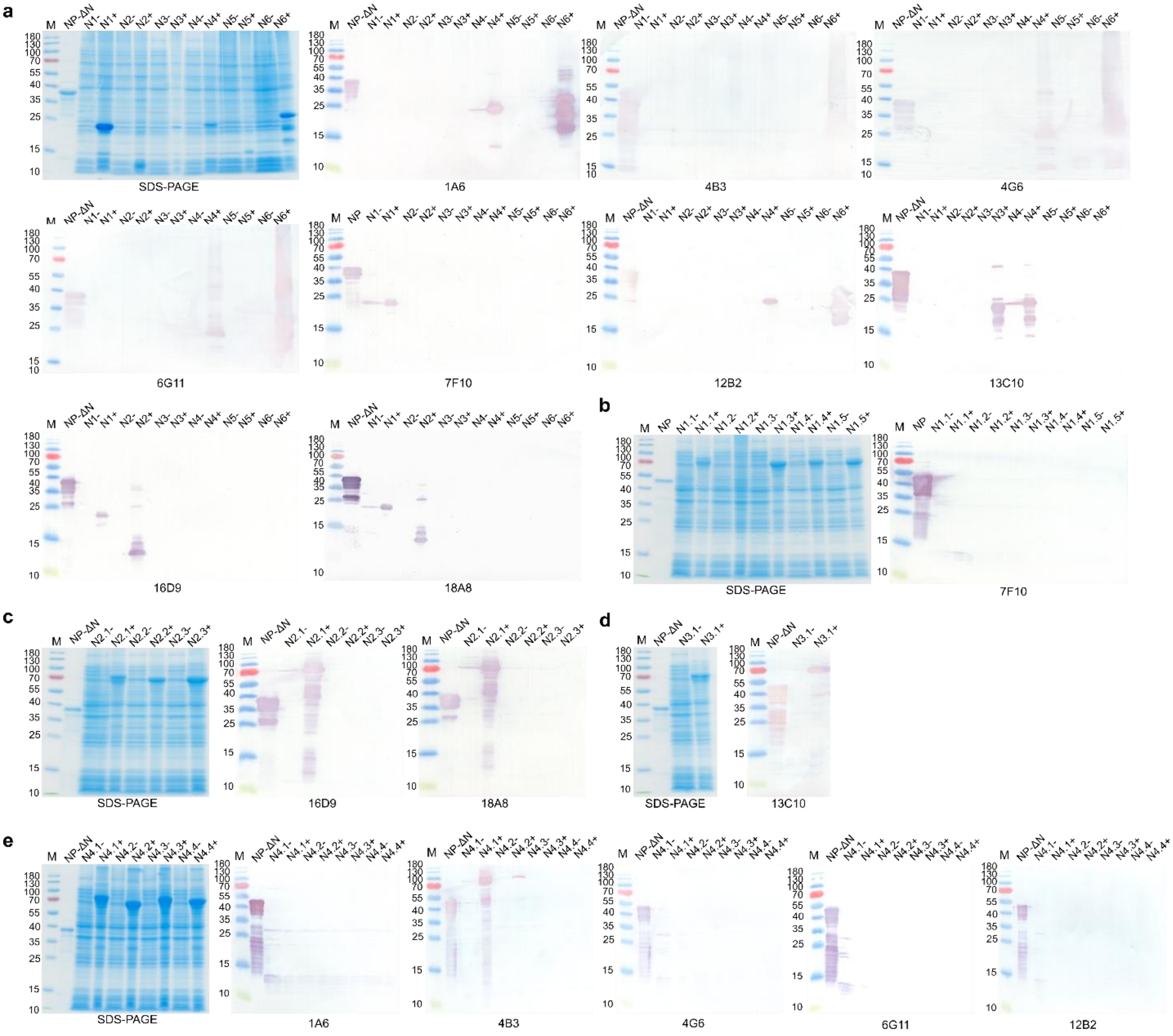

Fig S5. Western blot analysis for epitope mapping of anti-NP MAbs using overlapping and truncated NP fragments expressed in *E. coli*. Lysates containing overlapping NP fragments (1-6), subjected to WB and incubated with individual anti-NP MAb to map its binding region. Recombinant full-length NP and NP-ΔN served as positive controls. "–" and "+" indicate *E. coli* lysates collected before and after IPTG induction of protein expression, respectively (a). Epitope mapping was further explored using truncated NP fragments: MAb 7F10 with N1.1-N1.5 (b), MAbs 16D9 and 18A8 with N2.1-N2.3 (c), MAb 13C10 with N3.1 (d), and MAbs 1A6, 4B3, 4G6, 6G11, and 12B2 with N4.1-N4.4 (e). M – protein molecular weight marker (PageRuler™ Prestained Protein Ladder, 10-180 kDa, Thermo Scientific).

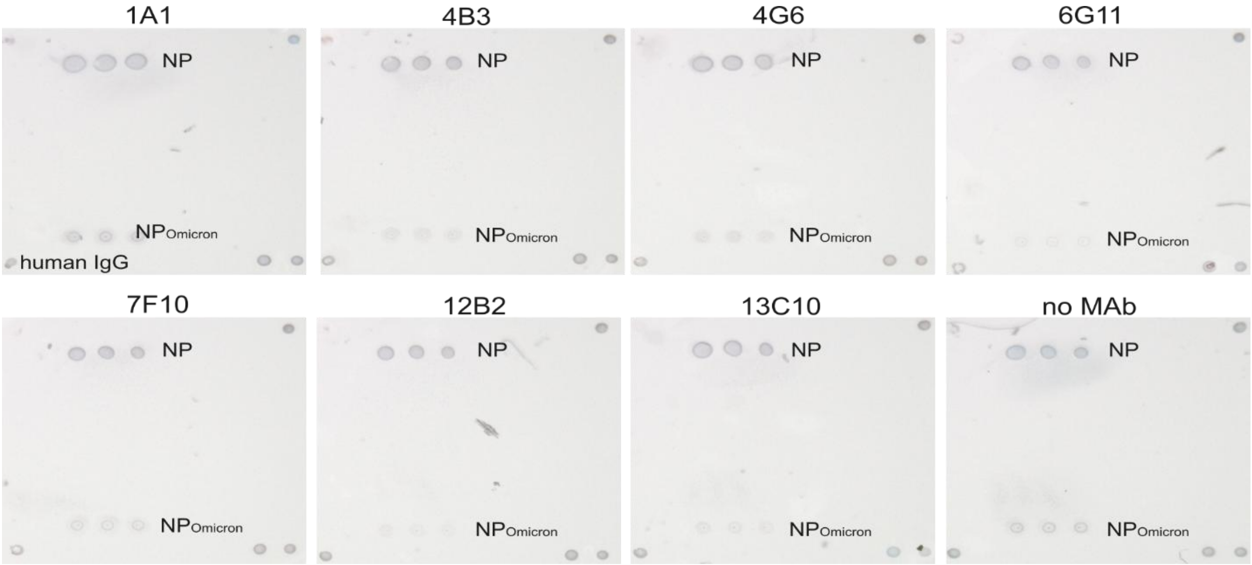

Fig S6. Microarray ELISA analysis illustrating no inhibition of human anti-SARS-CoV-2 NP IgG (sample #3) binding to the antigen in the presence of SARS-CoV-2 NP specific MAbs. The top three dots represent printed NP, and the bottom dots – $NP_{Omicron}$. Five control dots of human IgG were used in the corners of the microarray.
